# Genetic associations and candidate functional genes linking depression and obesity: a multi-omics integrative study

**DOI:** 10.3389/fgene.2026.1855173

**Published:** 2026-08-07

**Authors:** Xingpei Li, Chunlin Chen, Huibing Li, Yiru He, Kailang Tang, Guanqiao Lai, Ziyang Yang, Wushu Chen, Huihui Yang

**Affiliations:** 1 Department of Ultrasound Medicine, Guangdong Provincial Key Laboratory of Major Obstetric Diseases, Guangdong Provincial Clinical Research Center for Obstetrics and Gynecology, The Third Affiliated Hospital, Guangzhou Medical University, Guangzhou, China; 2 Department of Neurosurgery, Guangdong 999 Brain Hospital, Guangzhou, Guangdong, China; 3 Guangdong Provincial People's Hospital (Guangdong Academy of Medical Sciences), Southern Medical University, Guangzhou, China

**Keywords:** genetic association, *in silico* validation, major depressive disorder, mendelian randomization, multi-omics integration, obesity

## Abstract

**Introduction:**

Major depressive disorder (MDD) and obesity are intersecting global crises. Despite observational links, a clinical paradox persists: antidepressants often improve metabolic status, while weight loss rarely alleviates core depressive symptoms. This prompts closer examination of whether the depression–obesity relationship reflects asymmetric genetic architecture, shared liability, or statistical constraints that obscure definitive conclusions.

**Methods:**

We developed an integrative multi-omics framework leveraging large-scale population data from the National Health and Nutrition Examination Survey (NHANES) and East Asian genetic data. Epidemiological regression was applied to NHANES to characterize real-world phenotypic cross-talk. We utilized bidirectional Mendelian randomization (MR) to explore the direction of association, targeted summary-data-based MR (SMR) with heterogeneity in dependent instruments (HEIDI) testing to prioritize candidate functional genes, and single-cell RNA sequencing (scRNA-seq) of regulatory T cells (Tregs). In silico cell composition adjustment and virtual knockout (VKO) simulations were implemented to distinguish intrinsic cellular remodeling from compositional shifts and to infer convergent downstream programs.

**Results:**

Bidirectional MR yielded a nominally significant association from MDD to obesity risk (β = 0.0458, P = 0.0209), whereas the reverse path was inconclusive due to low statistical power (<10%), precluding definitive conclusions about directionality. SMR/HEIDI identified multiple FDR-significant obesity-associated genes, including NT5C2, ACYP2, and TMEM180, whereas on the depression side only ACAT1 reached nominal significance, positioning it as a borderline hypothesis-generating candidate. Cell composition adjustment suggested that transcriptional signals reflected intrinsic remodeling, preserving up to 98% of effect sizes for top candidates. At the molecular level, the conditions diverged: obesity risk was dominated by immune-compartment inflammation and post-transcriptional splicing dysregulation, whereas MDD risk was characterized by ribosomal translation perturbations. Strikingly, VKO simulations revealed convergence on a shared downstream program anchored in cytoskeletal reorganization and E2F-target modulation. Exploratory druggability screening nominated FDFT1 (with a phase 3 inhibitor) and ADORA2A as potential repurposing candidates requiring experimental validation.

**Conclusion:**

Our findings provide a hypothesis-generating reframing of the traditional comorbidity model, suggesting that divergent molecular programs may converge on shared pathways. Although the full extent of bidirectional genetic relationships remains unconfirmed, these findings offer a preliminary foundation for exploring therapeutic strategies at the mood–metabolism interface.

## Introduction

Major depressive disorder (MDD) and obesity represent two of the most formidable global public health crises of the 21st century, collectively contributing to an unprecedented proportion of the global non-fatal disease burden (Global, 2022; GBD 2021 Diseases and Injuries Collaborators, 2024). Despite their profound phenotypic convergence, and although decades of observational research have firmly established evidence supporting a bidirectional relationship, the underlying biological hierarchy governing this nexus remains mechanistically elusive (Pan et al., 2012). In clinical practice, an intriguing ‘tension’ has emerged: while ameliorating depressive symptoms frequently leads to spontaneous improvements in metabolic status, weight loss interventions alone often prove insufficient to alleviate core depressive symptoms (Pillinger et al., 2025; Osweiler et al., 2025; Goessl et al., 2023).

To date, several mechanistic hypotheses have been proposed to explain the intricate cross-talk at the mood-metabolism interface (Milaneschi et al., 2019). Chief among these is the hypothesis of shared chronic low-grade systemic inflammation, wherein elevated pro-inflammatory cytokines disrupt both central monoaminergic neurotransmission and peripheral lipid metabolism (Björntorp, 2001). Additionally, chronic stress-induced hyperactivation of the hypothalamic-pituitary-adrenal (HPA) axis represents a major convergent pathway, as sustained hypercortisolemia promotes central fat deposition while simultaneously inducing neuroinflammation (Björntorp, 2001). Neuroendocrine dysregulation, particularly involving leptin and insulin resistance, further bridges the two pathologies by impairing both homeostatic metabolic circuits and mood regulation (Hryhorczuk et al., 2013). Emerging clinical and genetic studies have begun to document a potential heterogeneity within these pathways, suggesting that the obesity-related genetic effects on depression may vary by symptom subtype—such as the increased-appetite or atypical depression symptom—rather than reflecting a uniform bidirectional genetic liability (Lasserre et al., 2014; Milaneschi et al., 2017). Nonetheless, a definitive mapping of whether genetic liability to depression is associated with downstream metabolic burden, or *vice versa*, requires robust population-scale validation.

A primary barrier to resolving this biological hierarchy is that traditional observational studies and cross-sectional investigations inherently suffer from residual confounding, socio-demographic biases, and reverse causation (Milaneschi et al., 2019). For instance, classical epidemiological cohorts like the National Health and Nutrition Examination Survey (NHANES) are highly valuable for identifying robust phenotypic correlations, yet they lack the capacity to decouple intrinsic causal drives from environmental noise (Zhao et al., 2011). To fully capture both the categorical disease architecture and continuous phenotypic variations, an analytical shift toward genetically informed multi-omics frameworks for assessing directionality is fundamentally required.

In this work, we addressed this causal ambiguity by developing an end-to-end integrative multi-omics framework leveraging large-scale East Asian genome-wide association study (GWAS) summary statistics and tissue-specific eQTLs (Chin et al., 2024; Biddie et al., 2024; Bowden and Holmes, 2019; Davies et al., 2018; Steptoe and Frank, 2023; Zhan et al., 2025; Casanova et al., 2021). We utilized bidirectional Mendelian randomization (MR) to estimate direction-specific genetic evidence while accounting for pervasive confounding, allowing us to test whether the observed genetic signals reflect a directional influence under current epidemiological and power constraints. To bridge the gap between genome-wide association studies and functional candidate genes, we integrated Summary-data-based Mendelian Randomization (SMR) with the Heterogeneity in Dependent Instruments (HEIDI) test to prioritize candidate genes within tissue-specific regulatory axes (Guo et al., 2025; Zhu et al., 2016).

Beyond population-level associations, achieving cell-type-specific resolution requires integration with single-cell RNA sequencing (scRNA-seq) data (Zhang et al., 2025; Zhang et al., 2024; Bang et al., 2025). We focus on regulatory T cells (Tregs) as a salient interface between systemic inflammation and metabolic regulation (Shi et al., 2026). Tregs represent a biologically critical candidate in this context because prior studies have deeply implicated them in the pathophysiology of both conditions (Capuron et al., 2017). In obesity, visceral adipose tissue (VAT)-resident Tregs are heavily enriched under lean conditions to maintain immunological tolerance and suppress insulin resistance; however, chronic nutrient excess depletes and phenotypically destabilizes this population, unleashing chronic tissue inflammation (Feuerer et al., 2009). Concurrently, in neuropsychiatric disorders like MDD, reductions in peripheral Treg frequencies and compromised suppressive functions may be associated with elevated systemic inflammation and increased blood-brain barrier permeability, facilitating peripheral-to-central inflammatory trafficking (Grosse et al., 2016). Tregs thus represent a unique cellular hub where metabolic stress and mood-related immune dysregulation organically converge. Critically, to disentangle whether observed disease signals stem from shifts in cell-type abundance or intrinsic intracellular remodeling, we implemented a counterfactual reweighting framework to adjust for cell composition. Furthermore, to move beyond descriptive profiling, we employ *in silico* virtual cell experiments, including gene knockout simulations (Yang et al., 2023). By computationally perturbing single-cell gene regulatory networks, we test whether divergent molecular pathologies—such as post-transcriptional dysregulation in obesity versus altered protein synthesis in depression—converge on shared downstream programs.

In this study, we develop an end-to-end integrative multi-omics framework integrating bidirectional MR, transcriptome-wide association, scRNA-seq, compositional deconvolution, virtual knockout, and drug target prioritization to dissect the depression–obesity nexus. By leveraging large-scale East Asian GWAS summary statistics and tissue-specific eQTLs, we bridge population-level genetic association analysis with cellular-resolution validation. We identify candidate molecular relays and explore the systems-level stress responses that unify distinct upstream insults. Collectively, this strategy aims to provide a hypothesis-generating foundation for future precision interventions at the mood-metabolism interface.

By discriminating genetically robust causal anchors from actionable translational nodes, our findings offer a reframing of the traditional symmetric model of MDD-obesity comorbidity by highlighting how statistical power asymmetries limit definitive causal inference. Collectively, this comprehensive strategy aims to provide a hypothesis-generating framework for future investigation at the mood-metabolism interface, informing potential directions for precision interventions pending experimental validation. The present framework is designed to generate testable hypotheses rather than to establish definitive directional causality.

## Methods

### Cross-sectional analysis of the depression–adiposity relationship in NHANES

Obesity is used to denote the clinically defined, categorical disease states established via strict diagnostic thresholds (GBD 2021 Diseases and Injuries Collaborators, 2024). Adiposity traits are used to refer to the underlying continuous phenotypic continuum of fat mass accumulation and distribution (modeled as continuous scores of BMI and waist circumference), which captures sub-clinical and broader population-level variations (Global et al., 2016). We analysed public-use data from seven continuous cycles of the NHANES (2005–2018) (Johnson et al., 2013). Non-pregnant adults aged 20 years or older were included if they had complete data for the Patient Health Questionnaire-9 (PHQ-9), measured body mass index (BMI), waist circumference, core demographic covariates (age, sex, race/ethnicity, education, smoking status, poverty-income ratio), and valid complex-survey design variables (SDMVPSU, SDMVSTRA). The final analytic sample comprised 15,488 participants. Clinically relevant depressive symptoms were defined as a PHQ-9 total score ≥10 (Kro et al., 2001), depressive symptom severity was additionally modelled as a continuous exposure per 5-point increment in PHQ-9 score. General obesity was defined as BMI ≥30 kg/m^2^ (Obesity: preventing and managing the, 2000), and abdominal obesity as waist circumference >102 cm in men and >88 cm in women (Lean et al., 1995). All analyses accounted for the NHANES complex multistage sampling design. Pooled 14-year examination weights were calculated as WTMEC2YR/7. Survey-weighted logistic regression was used to estimate odds ratios (ORs) for obesity and abdominal obesity, and survey-weighted linear regression was used to estimate β coefficients for continuous BMI and waist circumference.

Multivariable models were adjusted for age, sex, race/ethnicity, education, smoking status, poverty-income ratio, survey cycle, and relevant medication use (including antidepressants, glucose-lowering drugs, lipid-lowering agents, and anti-obesity medications). To further ensure the robustness of our findings against treatment-induced confounding, an additional sensitivity analysis was conducted by completely excluding individuals actively using any of these medications. Weighted descriptive analyses were performed across depressive symptom categories.

### Bidirectional Mendelian randomization

Depression and obesity GWAS summary statistics were integrated at the summary-statistics level, not by pooling individual-level data or simply concatenating raw datasets. For each trait, we first harmonized chromosome, base-pair position, effect allele, non-effect allele, effect size, standard error, allele frequency, and P value across all available GWAS datasets. Variants were aligned to a common allele direction using the 1000 Genomes Project Phase 3 reference panel. Duplicate or incompatible variants were removed, and meta-derived association estimates were generated from the harmonized summary statistics. For depression, five East Asian GWAS were included; for obesity, three East Asian GWAS were included, and all studies used consistent phenotype definitions as originally defined in the respective GWAS (i.e., we used the disease/trait status as provided by the original summary statistics without individual-level redefinition). Summary statistics for depression and obesity were derived from East Asian ancestry GWAS datasets. For MR instrument selection, independent variants were LD-clumped at r2 < 0.001 within a 10-Mb window using the 1000 Genomes Project Phase 3 reference panel as implemented in TwoSampleMR/OpenGWAS; the original implementation did not explicitly specify the East Asian subset (Abecasis et al., 2012). We then evaluated the retained MR instruments using instrument strength (F statistic (β^2^/SE^2^), with F > 10 indicating no weak instrument bias (Burgess et al., 2011)) and assessed meta-analytic heterogeneity across the contributing GWAS using Cochran’s Q and I^2^. All retained instruments had F > 10. Causal estimates were obtained using inverse-variance weighted (IVW) as the primary method, with weighted median, MR-Egger, simple mode, and weighted mode as sensitivity analyses (Davey Smith and Hemani, 2014; Bowden et al., 2016; Bowden et al., 2015). Robustness was further assessed using Cochran’s Q heterogeneity tests, MR-Egger intercepts, single-SNP analysis, leave-one-SNP-out analysis, and MR-PRESSO. Post-hoc power calculations were performed using a normal approximation based on the observed IVW β and its standard error, assuming α = 0.05. For each direction, we calculated (i) the power to detect the observed effect size (using its own β and SE), (ii) the power to detect the effect size observed in the opposite direction, and (iii) the minimum detectable absolute effect size (β) at 80% power. The approximate variance explained by the instruments (R^2^) was derived from allele frequencies and effect sizes. MR analyses were implemented mainly with the TwoSampleMR (Hemani et al., 2018) and MRPRESSO (Verbanck et al., 2018) packages in R, and nominal findings were interpreted conservatively because only two prespecified directions were tested.

### SMR and HEIDI analysis

To prioritize genes underlying the observed associations, we performed targeted Summary-data-based Mendelian Randomization (SMR) with the HEIDI test using the official SMR software (v1.3.1) (Zhu et al., 2016; Wu et al., 2018). For targeted SMR/HEIDI analyses, PsychENCODE PEER50 brain eQTL and CAGE lite blood eQTL resources were used in hg19/GRCh37 coordinates. Depression is primarily a neuropsychiatric disorder, and brain eQTL represent the most biologically relevant regulatory resource. For obesity, large-scale East Asian adipose or liver eQTL are not publicly available; blood eQTL from the CAGE consortium (blood, hg19) serve as a practical proxy that captures systemic inflammatory and metabolic signals relevant to obesity. The LD reference for SMR/HEIDI was based on the 1000 Genomes Project European Phase 3 panel, and only variants present in both the GWAS and eQTL summary statistics were retained (Abecasis et al., 2012). For each SMR-prioritized gene, we defined the SNP pair as the SMR top eQTL SNP and the regional GWAS lead SNP (the genome-wide significant GWAS variant with the smallest P value within ±1 Mb of the eQTL SNP). Pairwise LD (r^2^) was queried via the Ensembl REST API using the 1000 Genomes Phase 3 reference panel, summarizing EUR (CEU, FIN, GBR, IBS, TSI) and EAS (CDX, CHB, CHS, JPT, KHV) subpopulations separately. Benjamini–Hochberg false discovery rate (FDR) correction was applied across all probes tested within each trait. Genes with FDR <0.05 and HEIDI P > 0.01 were prioritized as robust candidates, whereas nominal SMR associations (P_SMR <0.05) with acceptable HEIDI support were retained as borderline, hypothesis-generating signals.

All eQTL datasets were used as pre-computed summary statistics; no individual-level data were accessed.

### Single-cell RNA-sequencing and pseudo-bulk analysis

Single-cell data from GSE261178 (obesity) and GSE308075 (depression) were used for pseudo-bulk differential expression, pathway reanalysis, candidate-gene follow-up, and exploratory network analyses. Seurat was used for single-cell object handling and visualization (Butler et al., 2018), and candidate-gene follow-up focused on pseudo-bulk expression trends, detection rates, and cell-level expression patterns. For pseudo-bulk analyses, counts were aggregated at the sample level within the relevant cell groups to generate pseudo-bulk count matrices (Crowell et al., 2020; Lun et al., 2016; Murphy and Skene, 2022). Expression values were normalized to logCPM with edgeR and differential expression was modeled with limma (Ritchie et al., 2015; Baldoni et al., 2025). To distinguish transcriptional changes driven by shifts in immune cell composition from those reflecting intrinsic cell-state remodeling, we adjusted for CD45 expression in the obesity dataset. Specifically, we performed overall analyses with CD45 as a covariate and conducted CD45-stratified analyses (CD45^+^ vs. CD45^−^) as a sensitivity check. In the depression dataset, Treg pseudo-bulk profiles were compared between MDD and control groups without additional covariate adjustment. Genes with FDR <0.05 were considered differentially expressed.

Pathway reanalysis was performed using fgsea (Subramanian et al., 2005) and GSVA (Hänzelmann et al., 2013) with Hallmark, Reactome, and KEGG gene sets; significance was defined by FDR <0.05 (Reimand et al., 2019; Geistlinger et al., 2021). Exploratory co-expression analysis used WGCNA, whereas TF activity/network summaries, cell composition analysis, and single-cell expression validation were treated as supportive analyses rather than primary inference (Langfelder and Horvath, 2008). Given the tissue imbalance in GSE261178, obesity-side single-cell-derived analyses were interpreted as exploratory evidence.

### Transcriptional validation of SMR-identified candidates

To provide expression-level support for SMR-prioritized candidates, we examined pseudo-bulk expression trends, detection rates, and cell-level expression patterns in the corresponding single-cell RNA-seq datasets. Obesity-side candidates were evaluated using the GSE261178 dataset, whereas depression-side candidates were assessed in Treg pseudo-bulk profiles from GSE308075. We focused on whether each candidate was reliably detected in the relevant cell population and whether its expression trend in pseudo-bulk analysis was directionally consistent with the disease comparison. These analyses were interpreted as exploratory expression-level follow-up rather than confirmatory validation, as none of the candidate-level expression differences remained significant after multiple-testing correction.

### Drug target and tractability assessment

To assess the translational potential of prioritized genes, we performed drug target profiling using the Open Targets Platform (v24.09) (Koscielny et al., 2017). The analysis focused on candidate genes identified from the SMR/HEIDI analysis and the top-ranked expression signals from the depression single-cell analysis. For the depression single-cell dataset, Treg cells were aggregated into sample-level pseudo-bulk profiles from 4 controls and 6 MDD cases. Differential expression was modeled using limma on edgeR-normalized logCPM values, with the contrast defined as MDD versus control. Positive logFC values indicate higher expression in MDD, whereas negative logFC values indicate lower expression in MDD (control as the reference group). P values were adjusted using the Benjamini–Hochberg method. This rank-based cutoff was applied consistently across analyses. This top 60 threshold was chosen pragmatically as a brief rationale to balance comprehensive pathway coverage with logistical downstream feasibility, rather than acting as a strict biological boundary. Consequently, the resulting druggability assessments are inherently threshold-dependent and should be explicitly regarded as exploratory and hypothesis-generating. For these selected genes, we extracted the number of known drugs, the maximum clinical phase achieved (approved, phase 4, phase 3, phase 2, phase 1, or preclinical), small-molecule tractability (structural or pocket-level), and representative drugs with their mechanisms of action. Based on these metrics, we prioritized genes with approved or phase 3 agents as actionable translational nodes and constructed gene–drug networks to visualize potential repurposing opportunities.

### In silico virtual cell experiments

To assess whether observed transcriptional signals reflected intrinsic cell-state remodeling rather than altered cell composition, and to test whether candidate genes from the two diseases converge on shared downstream programs, we performed two complementary *in silico* virtual cell experiments.

### Cell composition adjustment

For each dataset, cell subpopulations were defined based on clustering annotations: Treg subclusters for depression, and CD45-positive versus CD45-negative compartments for obesity (Sun et al., 2024; Hildreth et al., 2021). For each candidate gene, we reconstructed counterfactual expression profiles by reweighting the disease group’s subpopulation-specific expression levels to match the cell composition of the control group. The difference between the observed disease–control difference and the composition-adjusted estimate was interpreted as the within-state component, whereas the remaining difference was interpreted as the compositional component. Robustness was assessed by sample-level bootstrap resampling with 400 iterations (Cao et al., 2019).

### Virtual gene knockout

To investigate downstream convergence, we performed *in silico* gene knockout using scTenifoldKnk (Osorio et al., 2022). Background gene regulatory networks were constructed from control cells in each dataset: lean/control cells from GSE261178 for obesity and control Treg cells from GSE308075 for depression. Candidate genes from each disease side were individually perturbed, and significantly dysregulated downstream genes were identified using package-derived adjusted P values. Overlaps between knockout-responsive gene sets were evaluated through over-representation analysis of Hallmark, Reactome and KEGG pathways to identify shared downstream programs. Owing to tissue imbalance in the obesity single-cell dataset, obesity-side virtual knockout results were considered exploratory and hypothesis-generating. To assess whether observed overlaps were greater than expected by chance, we performed a specificity analysis using the hypergeometric test. For each cross-disease knockout pair, the expected number of shared downstream genes under the null hypothesis of independence was calculated based on the hypergeometric distribution. The observed/expected ratio and hypergeometric P value were then computed, and the false discovery rate (FDR) was controlled with the Benjamini–Hochberg method.

### Data and software availability

Summary statistics for depression were derived from five East Asian ancestry GWAS (GCST90435447, GCST90435440, GCST90435439, GCST90018613, and GCST006644) that were combined into a single dataset. For obesity, summary statistics from three East Asian ancestry GWAS datasets (GCST90477436, GCST90566414, and GCST90566416) were used, all defined using the obesity phenotype. Single-cell RNA-seq data for obesity (GSE261178) and depression (GSE308075) were obtained from GEO. NHANES data were obtained from the public-use files of seven continuous cycles (2005–2018), available via the National Center for Health Statistics. For SMR analysis, cis-eQTL data were obtained from PsychENCODE (brain, PEER50 normalized) for depression and from the CAGE consortium (blood, hg19) for obesity. Linkage disequilibrium reference was based on the 1000 Genomes Project Phase 3 panel. All analyses were performed in R (v4.2.0) using the following packages: TwoSampleMR (v0.7.0), MRPRESSO (v1.0), Seurat (v5.4.0), edgeR (v4.8.0), limma (v3.66.0), fgsea (v1.36.0), GSVA (v2.4.1), WGCNA (v1.74), scTenifoldKnk (v1.0.3), dplyr, and ggplot2. Targeted SMR/HEIDI analysis was performed using the standalone SMR software (v1.3.1). Druggability information was retrieved from the Open Targets Platform (v24.09).

## Results

### Depressive symptom burden is cross-sectionally associated with elevated adiposity

**FIGURE 1:**
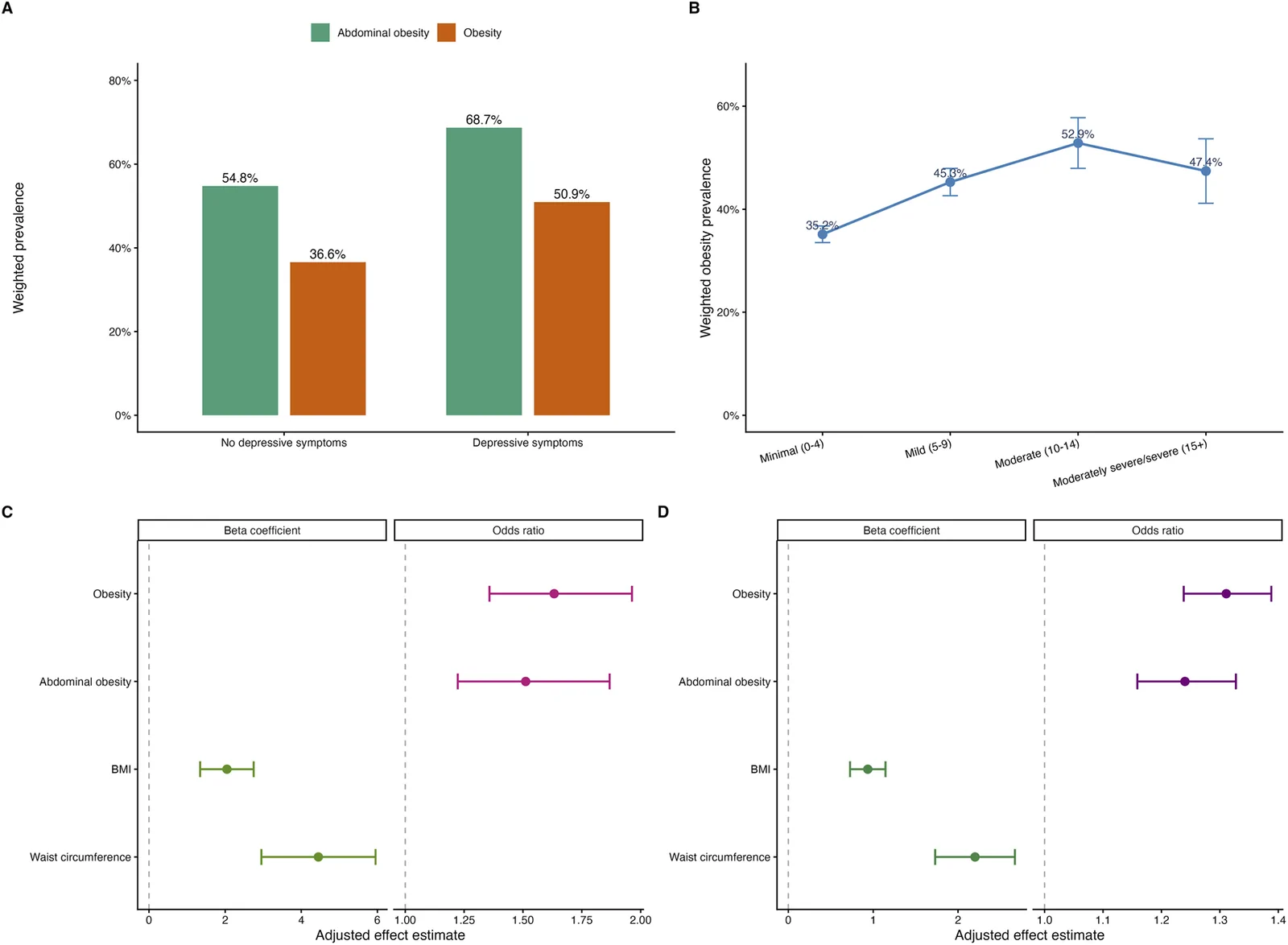
NHANES-based clinical validation of the association between depressive symptoms and obesity burden. **(A)** Weighted prevalence of obesity and abdominal obesity according to depressive symptom status defined by PHQ-9 ≥10. **(B)** Weighted obesity prevalence across PHQ-9 severity categories, showing a symptom-burden gradient. **(C)** Adjusted associations of depressive symptom positivity with obesity, abdominal obesity, BMI, and waist circumference in survey-weighted multivariable models. **(D)** Adjusted associations of each 5-point increase in PHQ-9 score with the same obesity-related outcomes. All models were adjusted for age, sex, race/ethnicity, education, smoking status, poverty-income ratio, and survey cycle, with full incorporation of the NHANES complex sampling design.

In the fully adjusted survey-weighted models within the NHANES cohort (n = 15,488), the presence of clinically relevant depressive symptoms (PHQ-9 ≥ 10) was associated with significantly higher odds of general obesity (OR = 1.39, 95% CI = 1.14–1.69, *P* < 0.001) and abdominal obesity (OR = 1.35, 95% CI = 1.09–1.67, *P* = 0.006). Depressive symptom positivity corresponded to an absolute increase of 1.43 kg/m^2^ in BMI (*P* < 0.001) and 2.95 cm in waist circumference (*P* < 0.001) (Figure 1). Furthermore, sensitivity analysis restricting the sample to non-medication users yielded highly consistent results, with the positive gradient between depressive symptoms and adiposity traits remaining statistically significant (all *P* < 0.05; Supplementary Figures S4, S5).

### Bidirectional MR provided nominal evidence for depression-to-obesity, but not the reverse

**FIGURE 2:**
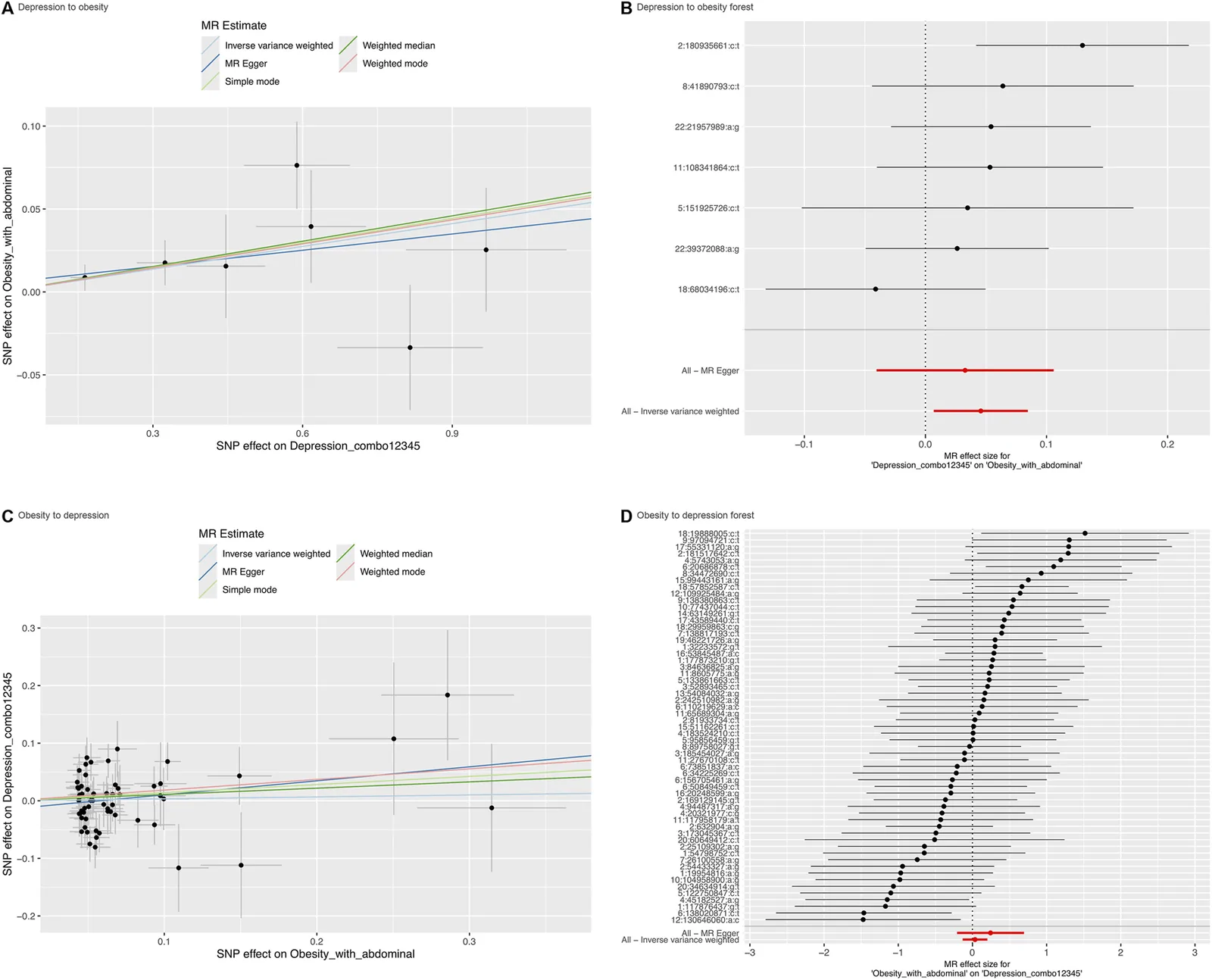
Bidirectional Mendelian randomization analysis between depression and obesity. **(A)** Scatter plot of SNP-specific effects for genetically predicted depression on obesity, with causal estimates from inverse-variance weighted (IVW), MR-Egger, weighted median, simple mode, and weighted mode methods. **(B)** Forest plot showing single-SNP and overall MR estimates for the depression-to-obesity direction. **(C)** Scatter plot of SNP-specific effects for genetically predicted obesity on depression. **(D)** Forest plot showing single-SNP and overall MR estimates for the obesity-to-depression direction. Together, these panels summarize the primary bidirectional MR framework and the consistency of direction-specific instrumental-variable estimates.

For the effect of depression on obesity, seven SNPs were selected as instrumental variables, with an average F-statistic of 32.25, indicating no weak instrument bias. The IVW method yielded a nominally significant positive association, where genetic predisposition to depression was associated with an increased risk of obesity (β = 0.0458, SE = 0.0198, *P* = 0.0209) (Figures 2A,B). The weighted median method corroborated this finding (β = 0.0510, SE = 0.0236, P = 0.0307). Sensitivity analyses revealed no evidence of heterogeneity (Cochran’s Q, *P* = 0.278, per-SNP contributions are detailed in Supplementary Table S2a) or horizontal pleiotropy (MR-Egger intercept *P* = 0.689, per-SNP contributions are detailed in Supplementary Table S2b). In contrast, the reverse analysis investigating the effect of obesity on depression included 59 SNPs. The IVW estimate provided non-significant for the effect of obesity on depression (β = 0.0342, SE = 0.0850, *P* = 0.688) (Figures 2C,D). However, significant heterogeneity was detected among the SNP estimates (Cochran’s Q, *P* = 0.024) (Figure 2D) (Table 1). The median I^2^ across instruments was 29% for depression and 59% for obesity (Supplementary Figure S2). Post-hoc power analysis showed that the reverse analysis had very low statistical power (observed-effect power for IVW <10%; see Supplementary Table S1; Supplementary Figure S1), indicating that the null result is uninformative and cannot be interpreted as evidence for or against a causal effect.

**TABLE 1:** Bidirectional MR summary.

Direction	nsnp	ivw b	ivw se	ivw p	weighted median b	weighted median p	egger b	egger p	ivw Q p	egger Q p	egger intercept	egger intercept p
Depression → Obesity	7	0.04575	0.01981	0.02094	0.05102	0.03070	0.03277	0.42002	0.27833	0.20444	0.00547	0.68924
Obesity → Depression	59	0.03416	0.08503	0.68789	0.10975	0.31782	0.24350	0.29476	0.02407	0.02473	−0.01402	0.33205

### SMR/HEIDI prioritized robust obesity genes and nominal depression candidates

**FIGURE 3:**
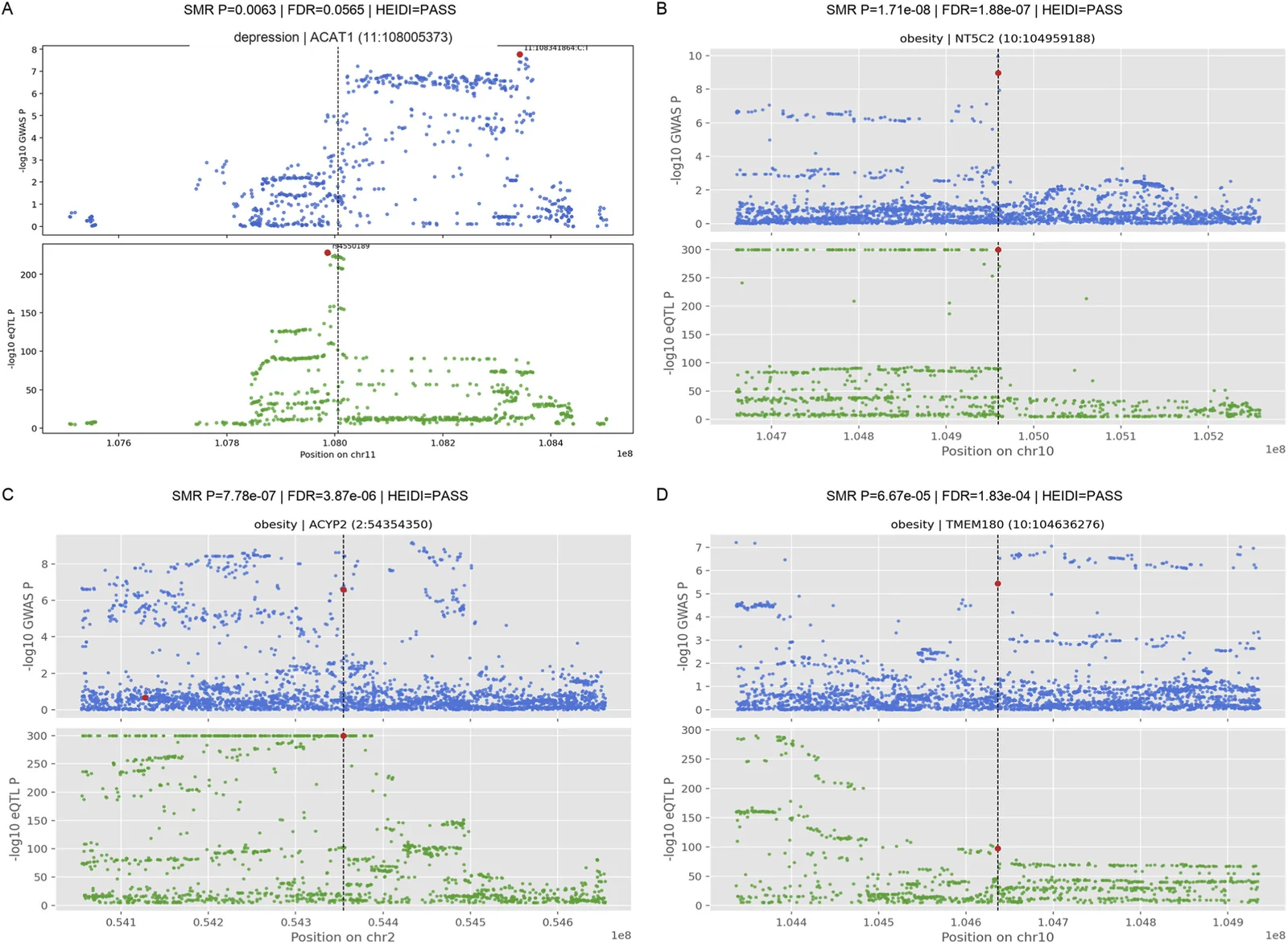
Colocalization-style regional association plots for prioritized SMR-supported loci. **(A)** Depression-associated locus centered on ACAT1. **(B)** Obesity-associated locus centered on NT5C2. **(C)** Obesity-associated locus centered on ACYP2. **(D)** Obesity-associated locus centered on TMEM180. For each locus, the upper panel shows GWAS association signals and the lower panel shows cis-eQTL association signals across the same genomic region. The vertical dashed line marks the lead variant, and the highlighted point indicates the focal variant used for regional interpretation. The panel titles summarize the corresponding SMR P value, FDR, and HEIDI result.

**FIGURE 4:**
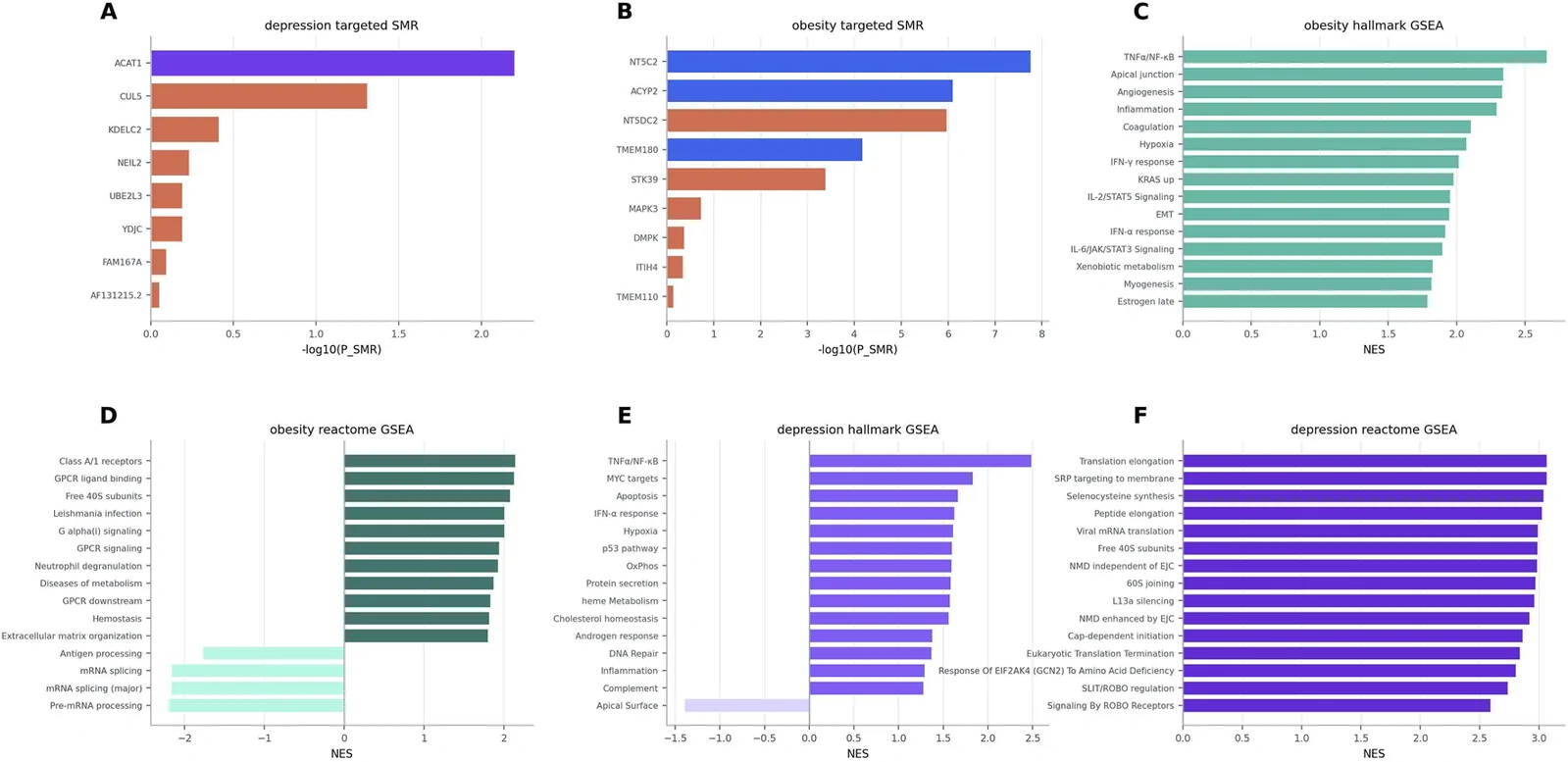
Integrated targeted SMR and pathway-level signals for depression and obesity. **(A)** Ranked targeted SMR signals for depression. **(B)** Ranked targeted SMR signals for obesity. **(C)** Hallmark GSEA results for obesity. **(D)** Reactome GSEA results for obesity. **(E)** Hallmark GSEA results for depression. **(F)** Reactome GSEA results for depression. Bar lengths represent signal magnitude, with SMR panels shown as −log10(P_SMR) and pathway panels shown as normalized enrichment scores (NES). This figure summarizes the main genetics-to-pathway axis of the study.

For obesity, five genes were significantly associated after FDR correction (FDR <0.05), three of which passed the HEIDI test (P_HEIDI > 0.01): *NT5C2* (P_SMR = 1.71 × 10^-8^), *ACYP2* (P_SMR = 7.78 × 10^−7^), and *TMEM180* (P_SMR = 6.67 × 10^−5^) (Figures 3B–D, 4B). Two additional genes—*NT5DC2* and *STK39*—were FDR-significant but did not pass the HEIDI test (P_HEIDI ≤ 0.01). For depression, nine genes were tested. *ACAT1* was nominally associated (P_SMR = 0.0063, FDR = 0.057) and passed the HEIDI test (P_HEIDI = 0.089) (Figures 3A, 4A), while *CUL5* was nominally significant (P_SMR = 0.049) but failed the HEIDI test (P_HEIDI = 0.004). No gene in the depression analysis was both FDR-significant and HEIDI-positive (Table 2). A sensitivity rerun using a single Japanese GWAS did not yield nominally significant SMR signals (Supplementary Table S3a).

**TABLE 2:** Official targeted SMR HEIDI priority.

Trait	Gene	topSNP	p GWAS	p eQTL	p SMR	smr fdr	p HEIDI	eqtl label
Depression	ACAT1	rs3741054	0.00184	1.20863 × 10^−8^	0.00628	0.05652	0.08924	PsychENCODE PEER50 brain eQTL
Depression	CUL5	rs7102368	0.03552	1.73585 × 10^−8^	0.04886	0.21987	0.00420	PsychENCODE PEER50 brain eQTL
Obesity	NT5C2	rs11191607	1.07645 × 10^−9^	1.04063 × 10^−49^	1.70891 × 10^−8^	1.87980 × 10^−7^	0.58945	CAGE lite blood eQTL (hg19)
Obesity	ACYP2	rs7592069	2.48362 × 10^−7^	4.55258 × 10^−66^	7.78418 × 10^−7^	3.86810 × 10^−6^	0.03560	CAGE lite blood eQTL (hg19)
Obesity	NT5DC2	rs13621	1.22900 × 10^−7^	8.30266 × 10^−37^	1.05494 × 10^−6^	3.86810 × 10^−6^	0.00437	CAGE lite blood eQTL (hg19)
Obesity	TMEM180	rs77335224	3.60543 × 10^−6^	4.74886 × 10^−15^	6.67197 × 10^−5^	0.00018	0.08283	CAGE lite blood eQTL (hg19)
Obesity	STK39	rs4668044	0.00026	8.77368 × 10^−46^	0.00041	0.00089	0.00072	CAGE lite blood eQTL (hg19)

Cross-population LD sensitivity analysis revealed locus-specific patterns (Supplementary Table S3c). Among the evaluable obesity-side loci, *NT5C2* showed highly similar LD between EUR and EAS panels (mean r^2^ = 1.000 vs 0.977), whereas *ACYP2* and *TMEM180* showed moderate-to-large differences (r^2^ difference = 0.166 and 0.269, respectively). For the depression-side candidate *ACAT1*, the lead eQTL-GWAS SNP pair lacked EAS LD reference data and was therefore not evaluable.

### Pathway reanalysis highlighted inflammatory and RNA-processing programs in obesity

Given that initial GO Biological Process analysis yielded unstable results, we performed a further pathway re-analysis using Hallmark, Reactome, and KEGG gene sets on the pseudo-bulk count matrix with adjustment for CD45. GSEA identified the Hallmark TNF-alpha signaling via NF-kB pathway as significantly enriched in obesity compared to lean controls (NES = 2.66, FDR = 6.55 × 10^−17^) (Figure 4C). Reactome analysis identified Processing of Capped Intron-Containing Pre-mRNA as the downregulated pathway (NES = −2.20, FDR = 1.39 × 10^-8^), while KEGG analysis showed Spliceosome as the most significantly enriched pathway (NES = −2.37, FDR = 2.66 × 10^-8^) (Figure 4D). Subgroup analysis restricted to CD45-positive cells further confirmed the upregulation of TNF-alpha signaling (logFC = 0.75, FDR = 0.004). CD45-stratified analysis revealed that the TNF-alpha signaling pathway was strongly enriched in CD45^+^ cells (NES = 3.13, FDR <0.001) but not in CD45^−^ cells (NES = −1.16, FDR = 0.32; Supplementary Table S5), indicating that the inflammatory signal originates primarily from the immune compartment.

### Pathway reanalysis in depression tregs supported inflammatory and translational dysregulation

As no genes reached FDR <0.05 in the pseudo-bulk analysis of Tregs from four controls and six MDD patients, we performed a further pathway re-analysis using Hallmark, Reactome, and KEGG gene sets. FGSEA identified TNF-alpha Signaling via NF-kB as the significantly enriched Hallmark pathway in MDD compared to controls (NES = 2.49, FDR = 5.17 × 10^−13^) (Figure 4E). Reactome analysis showed SRP-dependent Cotranslational Protein Targeting to Membrane as the significantly enriched pathway (NES = 3.07, FDR = 8.09 × 10^−18^), and KEGG analysis identified Ribosome as the significantly enriched pathway (NES = 2.62, FDR = 6.26 × 10^−12^) (Figure 4F). In total, 10 Hallmark, 182 Reactome, and 36 KEGG pathways were significantly enriched at FDR <0.05. In contrast, GSVA using the same gene sets did not yield significant results at FDR <0.05 (Table 3).

**TABLE 3:** Pathway core summary.

Trait	Analysis	Collection	Method	Pathways	fdr lt 0 05	Best pathway	Best score	Best padj
Depression	Overall mdd vs. control	Hallmark	fgsea	50	10	TNF-alpha Signaling via NF-kB	2.48981	5.17216 × 10^−13^
Depression	Overall mdd vs. control	Kegg	fgsea	301	36	Ribosome	2.61831	6.25601 × 10^−12^
Depression	Overall mdd vs. control	Reactome	fgsea	1255	182	SRP-dependent Cotranslational Protein Targeting To Membrane R-HSA-1799339	3.06696	8.09360 × 10^−18^
Obesity	Overall adjust cd45	Hallmark	fgsea	50	31	TNF-alpha Signaling via NF-kB	2.66046	6.55077 × 10^−17^
Obesity	Overall adjust cd45	Kegg	fgsea	307	119	Spliceosome	−2.37149	2.65560 × 10^−8^
Obesity	Overall adjust cd45	Reactome	fgsea	1319	144	Processing Of Capped Intron-Containing Pre-mRNA R-HSA-72203	-2.20487	1.38495 × 10^−8^
Obesity	Stratified CD45 negative	hallmark	fgsea	50	13	Interferon Alpha Response	2.44393	2.36234 × 10^−9^
Obesity	Stratified CD45 negative	Kegg	fgsea	305	4	Osteoclast differentiation	−1.83749	0.00983
Obesity	Stratified CD45 negative	Reactome	fgsea	1294	10	Interferon Alpha/Beta Signaling R-HSA-909733	2.35760	5.85944 × 10^−5^
Obesity	Stratified CD45 positive	Hallmark	fgsea	50	31	TNF-alpha Signaling via NF-kB	3.12850	1.42593 × 10^−30^
Obesity	stratified CD45 positive	Hallmark	gsva	50	11	TNF-alpha Signaling via NF-kB	0.75027	0.00403
Obesity	Stratified CD45 positive	Kegg	fgsea	303	150	Herpes simplex virus 1 infection	−2.17908	2.55675 × 10^−13^
Obesity	stratified CD45 positive	Reactome	fgsea	1277	171	Neutrophil Degranulation R-HSA-6798695	2.49781	1.39243 × 10^−20^

### Transcriptional follow-up of SMR-prioritized candidates

**FIGURE 5:**
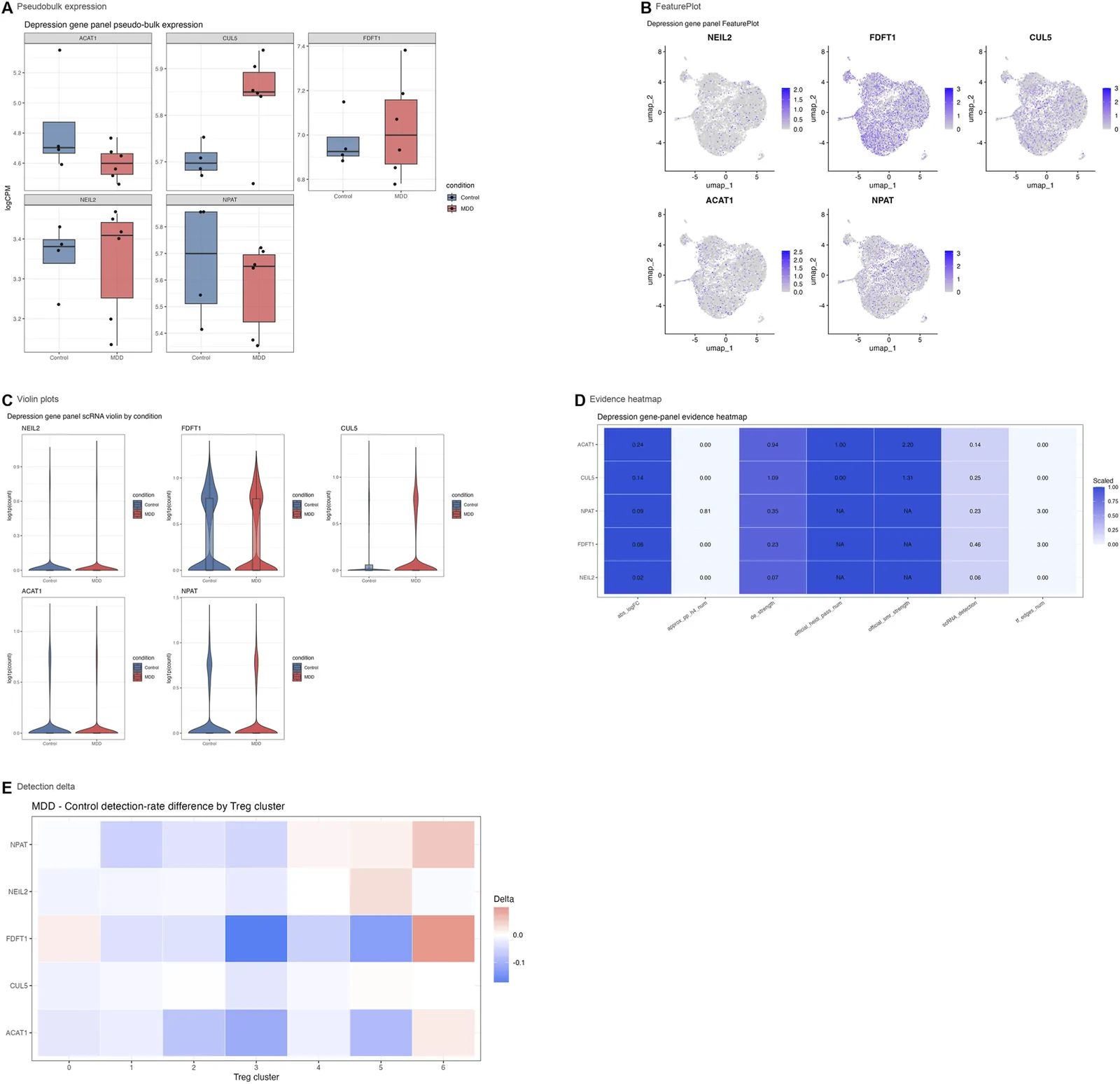
Expression-level follow-up of depression candidate genes in single-cell and pseudo-bulk analyses. **(A)** Pseudo-bulk expression of prioritized depression genes in control and MDD samples. **(B)** UMAP FeaturePlots showing the spatial distribution of candidate-gene expression across single cells. **(C)** Violin plots showing expression distributions by condition. **(D)** Integrated evidence heatmap summarizing genetics, expression, and follow-up support across depression candidate genes. **(E)** Heatmap of detection-rate differences across Treg subclusters between MDD and control samples. Together, these panels provide transcriptional support for prioritized depression-side candidates.

*ACYP2* showed an upward trend in obesity pseudo-bulk analysis (logFC = 0.213), whereas *NT5C2* showed little expression-level support (logFC = −0.043). *TMEM180* was not reliably detected and could not be robustly evaluated. On the depression side, *ACAT1* showed a downward trend (logFC = −0.235) together with a lower detection rate in MDD than in controls, whereas *CUL5* showed a mild upward trend (logFC = 0.135) with broadly similar detection rates between groups (Figures 5A–E). However, none of these candidate-level differences remained statistically significant after multiple-testing correction (Table 4).

**TABLE 4:** Depression GenePanel summary.

Gene symbol	Official p SMR	Official p HEIDI	Official smr fdr	de logFC	de FDR	Mean log1p expr control	Mean log1p expr MDD	Detection rate control	Detection rate MDD
ACAT1	0.00628	0.08924	0.05652	−0.23545	0.41121	0.09841	0.07258	0.14325	0.10250
CUL5	0.04886	0.00420	0.21987	0.13515	0.36345	0.18462	0.17622	0.25000	0.23683
NPAT				−0.09233	0.72165	0.17113	0.15343	0.23075	0.20217
FDFT1				0.06408	0.82001	0.36944	0.34859	0.46150	0.43400
NEIL2				−0.01845	0.94257	0.04315	0.03597	0.06150	0.05050

### *In silico* validation

#### Cell composition adjustment confirms within-state remodeling

**FIGURE 6:**
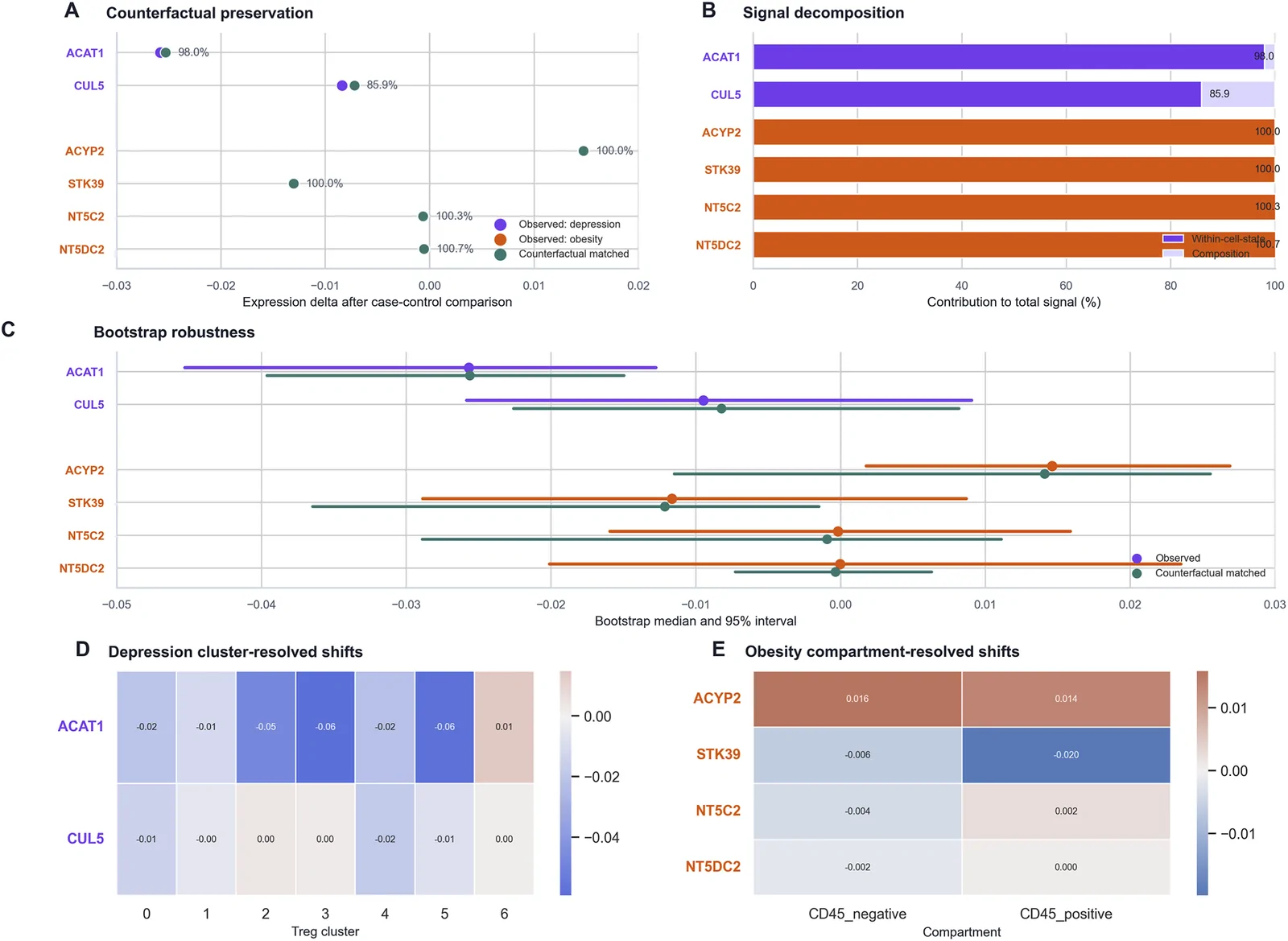
In silico virtual cell experiments support within-cell-state remodeling. **(A)** Dumbbell plot comparing the observed case-control expression delta with the counterfactual composition-matched estimate for each candidate gene. Labels indicate the percentage of the original signal retained after matching. **(B)** Decomposition of the total signal into within-cell-state and cell-composition components. Depression-side effects are dominated by within-cluster remodeling, whereas obesity-side effects are almost entirely preserved after CD45 balancing. **(C)** Sample-level bootstrap medians and empirical 95% intervals for observed and counterfactual deltas, showing that the matched estimates remain centered close to the observed effects. **(D)** Cluster-resolved expression shifts across depression Treg subclusters. **(E)** Compartment-resolved expression shifts across CD45-negative and CD45-positive obesity compartments.

In depression Tregs, adjusting Treg subcluster composition to match control distribution minimally altered the MDD compared with control expression difference for *ACAT1* (observed −0.0258 compared with composition-adjusted −0.0253), preserving 98.0% of the original effect. *CUL5* showed a similar pattern (85.9% preservation). In obesity, balancing CD45-positive compared with CD45-negative compartments yielded near-identical estimates for *ACYP2* (0.01473 compared with 0.01472) and *STK39* (−0.01306 compared with −0.01305) (Figures 6A,B). Bootstrap resampling confirmed robustness across iterations (Figures 6C–E).

#### Virtual knockout reveals convergent downstream programs

**FIGURE 7:**
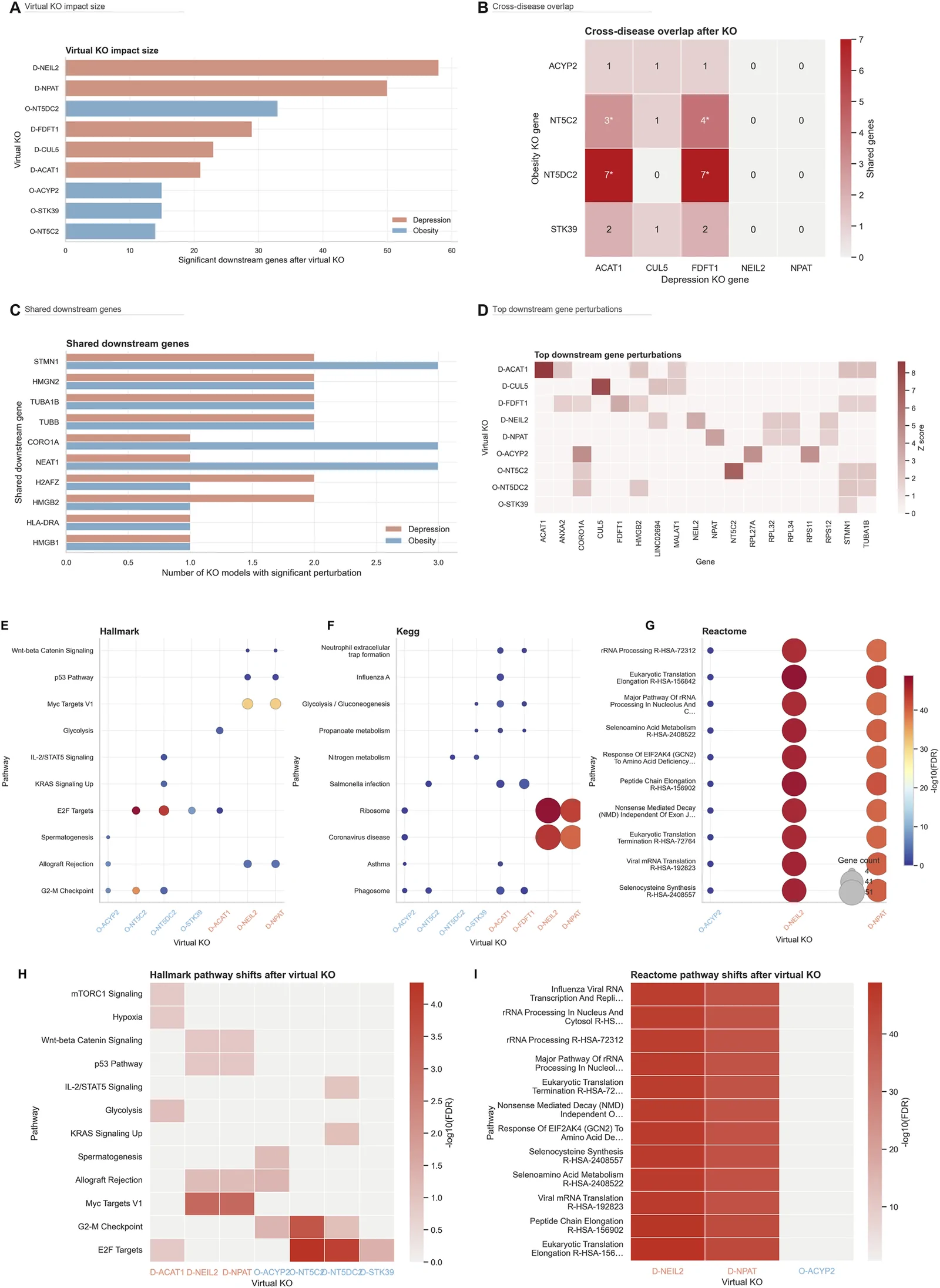
Integrated virtual knockout analysis of prioritized depression and obesity genes. **(A)** Overall virtual KO impact size measured by the number of significantly perturbed downstream genes. **(B)** Cross-disease overlap heatmap showing shared downstream perturbations between obesity and depression KO models. **(C)** Shared downstream genes recurrently perturbed across KO models. **(D)** Heatmap of top downstream gene perturbations across KO settings. **(E)** Hallmark pathway bubble plot after virtual KO. **(F)** KEGG pathway bubble plot after virtual KO. **(G)** Reactome pathway bubble plot after virtual KO. **(H)** Hallmark pathway-shift heatmap after virtual KO. **(I)** Reactome pathway-shift heatmap after virtual KO. These panels show that independent candidate-gene perturbations converge on shared downstream programs and pathway-level signatures.

Virtual knockout of candidate genes perturbed varying numbers of downstream genes. *NEIL2* and *NPAT* (depression side) and *NT5DC2* (obesity side) showed larger effects (Figure 7A). Cross-disease overlap analysis showed that knockout combinations involving obesity-side *NT5DC2*/*NT5C2* and depression-side *ACAT1*/*FDFT1* shared the most downstream genes (7 shared genes) (Figure 7B). Frequently shared nodes included *STMN1*, *CORO1A*, *NEAT1*, *TUBA1B*, *TUBB*, and *HMGN2* (Figures 7C,D). Pathway enrichment of the shared genes revealed significant enrichment in E2F Targets, G2-M Checkpoint, Myc Targets V1, Phagosome, and ribosome-related processes (Figures 7E–I). A hypergeometric-based specificity analysis was performed to evaluate whether the observed downstream overlaps could occur by chance (Supplementary Table S4; Supplementary Figure S3). For cross-disease knockout pairs with significant overlap, the observed number of shared downstream genes was 8.6- to 13.9-fold higher than the expected number under random independence. For example, the NT5DC2 × ACAT1 pair showed an observed/expected ratio of 13.14 (observed = 7, expected = 0.53, hypergeometric FDR = 5.8 × 10^−6^). These results indicate that the observed overlaps are not merely due to random set-size effects.

#### Drug target profiles of candidate genes prioritize *FDFT1*, *ADORA2A*, and *PDGFB*

**FIGURE 8:**
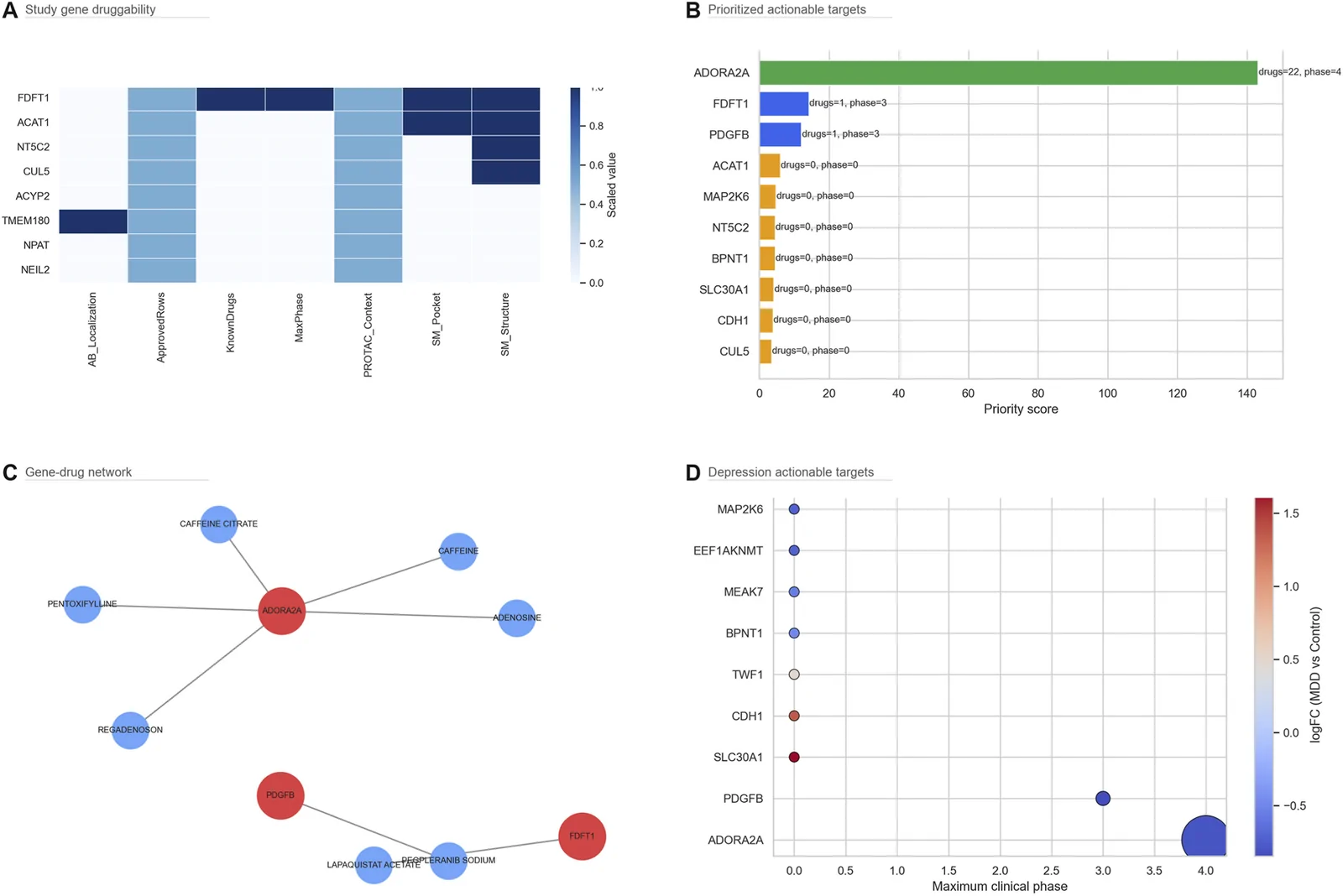
Drug-target and tractability assessment of prioritized study genes. **(A)** Druggability heatmap summarizing tractability-related features across candidate genes. **(B)** Ranked actionable targets according to composite prioritization scores. **(C)** Gene–drug interaction network linking prioritized genes with known compounds. **(D)** Bubble plot summarizing depression-relevant actionable targets by clinical-development maturity and transcriptional direction. This figure distinguishes putative mechanistic genes from genes with clearer translational or pharmacological potential.

Because no depression Treg pseudo-bulk gene passed BH FDR <0.05, we treated the top 60 nominally ranked genes from the MDD versus control comparison as an exploratory expression-supported set for druggability screening rather than as a formally significant DE gene set. We then queried the Open Targets Platform for candidate genes from the SMR analysis together with this exploratory set (Figure 8A). Among the SMR-identified genes, *FDFT1* had the most advanced clinical pipeline: its inhibitor, lapaquistat acetate, has reached phase 3 trials (Figure 8B). *ACAT1*, *CUL5*, and *NT5C2* showed small-molecule tractability (structural or pocket-level) but had no approved or investigational drugs. Among differentially expressed genes in depression, *ADORA2A* had the most known drugs (n = 22), including multiple phase 4 or approved agents. *PDGFB* was associated with a phase 3 drug (pegpleranib sodium) (Figures 8C,D) (Table 5). These nominations are threshold-dependent and should be considered hypothesis-generating.

**TABLE 5:** Drug target priority.

Gene symbol	Known drug unique	Max clinical phase	Priority score	Top drugs short	Top moas short
ADORA2A	22.00000	4.00000	143.19822	ADENOSINE | REGADENOSON | PENTOXIFYLLINE	Adenosine receptor agonist | Adenosine A2a receptor agonist | Adenosine A2 receptor antagonist
FDFT1	1.00000	3.00000	14.20000	LAPAQUISTAT ACETATE	Squalene synthetase inhibitor
PDGFB	1.00000	3.00000	12.04521	PEGPLERANIB SODIUM	Platelet-derived growth factor subunit B inhibitor
ACAT1	0.00000	0.00000	6.00000		
NT5C2	0.00000	0.00000	4.50000		
CUL5	0.00000	0.00000	3.50000		
ACYP2	0.00000	0.00000	3.00000		
TMEM180	0.00000	0.00000	3.00000		

## Conclusion

Our integrative multi-omics provides a hypothesis-generating reframing of the traditional bidirectional model of depression–obesity comorbidity. We found nominal genetic evidence for an association from depression to obesity, whereas the reverse pathway remained inconclusive owing to limited statistical power; thus, a definitive causal asymmetry cannot be established. Leveraging large-scale East Asian genetic data, we find suggestive evidence that depressive liability exerts a directional influence on metabolic burden, whereas the reverse effect was inconclusive. Through *in silico* cell composition adjustment, our *in silico* analyses suggest that these associations reflect intrinsic intracellular remodeling rather than shifts in cell-type abundance, supporting the biological plausibility of the identified candidate relays.

At the molecular level, the observed divergence is underpinned by distinct regulatory programs: depression is characterized by global translational dyshomeostasis and ribosomal enrichment within the immune compartment, whereas obesity is defined by systemic inflammatory activation and pervasive post-transcriptional splicing aberrations. Strikingly, despite these distinct upstream origins, virtual knockout experiments uncover a unifying systems-level stress response—anchored in cytoskeletal reorganization and phagosomal signaling—suggesting that disparate neuropsychiatric and metabolic insults may converge on shared downstream programs.

By discriminating genetically anchored candidates from actionable translational nodes, this study provides a hypothesis-generating molecular framework for future investigation. Collectively, these findings offer a preliminary framework linking symptomatic correlation to biological vulnerability, highlighting the potential value of early metabolic monitoring in depression as a hypothesis for future validation and providing a preliminary foundation for exploring concurrent therapeutic strategies at the mood–metabolism interface.

## Discussion

The intricate relationship between mental and metabolic health has long been framed by a model of bidirectional feedback, where each condition exacerbates the other (Pan et al., 2012; Opel et al., 2025; Pasman et al., 2025). Here, we re-examine this hypothesis by integrating large-scale bidirectional Mendelian randomization, cross-tissue transcriptome-wide association studies, and *in silico* cellular perturbation experiments. Our findings suggest a potential directional influence of MDD on obesity, as the forward Mendelian randomization analysis yielded a nominally significant effect. However, the reverse analysis was severely underpowered (power <10%), and its null result cannot be taken as evidence against a causal effect of obesity on depression. Thus, while our data are consistent with a model in which depression contributes to metabolic susceptibility, the observed pattern may reflect asymmetric statistical power rather than a true biological asymmetry; this interpretation, however, requires further investigation in better-powered studies.

Decades of observational and genetic research have accumulated evidence for a bidirectional relationship between obesity and depression (Pan et al., 2012; Opel et al., 2025; Pasman et al., 2025). However, the expansion of genome-wide association studies and the refinement of multivariable Mendelian randomization are now suggesting potentially nuanced directional patterns that warrant further investigation. Recent studies underscore this realignment: depression shows a robust association with type 2 diabetes, with 36.5% of this effect mediated by body mass index (Maina et al., 2023). Similarly, waist-to-hip ratio mediates 69% of the total effect of depression on triglyceride levels (Sun et al., 2025), highlighting central adiposity as a key factor in metabolic dysregulation associated with depression. The significant heterogeneity observed in the reverse analysis further suggests that the effect of obesity on depression is likely dispersed across multiple, heterogeneous biological pathways rather than driven by a single dominant mechanism.

This divergent associational pattern aligns with previous deep-dive studies on specific metabolic phenotypes: a positive association between depression and waist-to-hip ratio has been supported by previous MR evidence (Zhan et al., 2025), whereas the causal effect of obesity on depression appears to be driven only in specific subtypes—such as the increased-appetite symptom—rather than reflecting a uniform genetic liability (Pistis et al., 2021; Speed et al., 2019). This pattern suggests that the mood-lowering effects of obesity observed clinically may stem from secondary mechanisms rather than direct core genetic drivers.

The NHANES findings provide clinical context for this genetic divergence. Notably, adjusting for or completely excluding individuals taking antidepressants and metabolic medications did not alter the significant associations between depressive symptoms and adiposity traits, confirming the phenotypic overlap is independent of treatment-induced side effects. The robust, graded association between depressive symptom burden and adiposity mirrors the directionality of the forward MR signal, whereas the substantial heterogeneity in the reverse MR analysis suggests that the observational correlation from obesity to depression is unlikely to reflect a unified genetic pathway. Instead, it may represent a composite of heterogeneous mechanisms, including inflammation-mediated effects secondary to adiposity or residual confounding. A nominal sex difference—stronger associations in women—was noted in sensitivity analyses, echoing prior evidence of sex-specific genetic covariance, though dedicated sex-stratified MR was precluded by summary data resolution.

One such mechanism may involve inflammation. Genetic evidence shows that while C-reactive protein has no direct causal relationship with depression, body mass index is associated with higher C-reactive protein levels (Palmos et al., 2023). This supports a model in which is associated with higher obesity risk, which in turn may contribute to systemic inflammation. Consistent with this, our pathway analyses revealed that both obesity and depression share an enrichment of inflammatory pathways—not because depression directly triggers inflammation, but rather because the metabolic consequences of depression (i.e., obesity) propagate an inflammatory state that may contribute to the clinical overlap between the two conditions.

At the molecular level, our targeted SMR/HEIDI analysis revealed a divergent pattern of molecular signals between the two conditions. The metabolic phenotype presented a more defined gene landscape, in sharp contrast to the more diffuse signal on the depression side. On the obesity side, *NT5C2* emerged as the statistically most robust candidate, with evidence linking it to energy sensing and translational control through regulation of the intracellular adenylate pool (Moulin et al., 2026). Notably, *ACYP2*—which also passed rigorous genetic filtering—showed a trend toward differential expression in obesity pseudo-bulk analysis, positioning it as a prioritized candidate for functional follow-up. *ACYP2* encodes acylphosphatase 2, an enzyme involved in energy metabolism; its dysregulation could affect ATP turnover and mitochondrial function, potentially contributing to the metabolic disturbances observed in obesity (McGilvrey et al., 2025; Borns et al., 2020; Nesci and Rubattu, 2024). *TMEM180* was additionally identified as a conservative priority candidate with consistent local locus-level support, though its function remains poorly characterized (Wang et al., 2021; Waage et al., 2018).

On the depression side, only ACAT1 reached borderline significance with acceptable heterogeneity, making it a hypothesis-generating anchor for MDD-related metabolic dysregulation; *CUL5* was excluded from core candidates due to significant heterogeneity in the HEIDI test. ACAT1 encodes acetyl-CoA acetyltransferase 1, a key enzyme in ketone body metabolism and mitochondrial fatty acid oxidation. Its reduced detection rate in MDD Tregs hints at possible alterations in immune cell energy metabolism, which could link depressive states to systemic metabolic dysregulation. Although the genetic evidence for ACAT1 is only borderline, it provides a testable hypothesis for future functional studies.

Beyond these SMR-prioritized candidates, several genes identified through drug target prioritization represent translational entry points. *FDFT1* encodes squalene synthase and serves as the first committed enzyme in cholesterol biosynthesis. Rare deleterious variants in this gene cause severe neurodevelopmental deficits (Coman et al., 2018). Its dysfunction may also affect synaptic lipid composition (Oberst et al., 2026) or compromise myelin integrity (Barnes-Vélez et al., 2023). Notably, adult knockout studies suggest that glial compensation can maintain basal function under unstressed conditions (Fünfschilling et al., 2007). Inhibitors of this enzyme have been evaluated in clinical trials for lipid lowering (Bergstrom et al., 1993; Do et al., 2009), and statin use has been associated with reduced depressive symptoms (Gutlapalli et al., 2022). *ADORA2A* encodes the adenosine A2A receptor and represents a well-validated drug target, with approved antagonists including istradefylline, which is used for Parkinson disease and has been shown in clinical studies to reduce off time in Parkinson disease (Jenner, 2005; Berger et al., 2020; Hatano et al., 2024). The gene has extensive links to mood regulation (Wang et al., 2023; Yue et al., 2026; Bevilacqua et al., 2025; Pasquini et al., 2022) and peripheral metabolism (Verma et al., 2025; Kim et al., 2022), while its overactivation can lead to HPA axis dysfunction (Batalha et al., 2016). *PDGFB* (platelet-derived growth factor subunit B) has been evaluated in phase 3 trials (pegpleranib sodium; NCT01940887, NCT01940900) for ophthalmic indications, underscoring its druggability. These genes, while not prioritized by our genetic mapping, offer actionable nodes for repurposing or developing therapies targeting the interface between mood and metabolism (Figure 8C).

To further validate the robustness of these candidate gene signals and to test whether distinct genetic drivers converge on shared downstream mechanisms, we performed two complementary *in silico* validation approaches. First, by reweighting cell composition to control for potential confounding from altered cell-type proportions, we found that the expression shifts for depression-side candidates (ACAT1, CUL5) and obesity-side candidates (ACYP2, STK39) were largely preserved after composition adjustment, indicating that the transcriptomic signatures reflect intrinsic within-cell-state remodeling rather than secondary changes in cell abundance. Second, using virtual knockout simulations, we observed that despite the absence of direct overlap between depression-side and obesity-side candidate genes, their perturbations consistently converged on a common set of downstream genes, including STMN1, CORO1A, NEAT1, TUBA1B, TUBB and HMGN2, with pathway enrichment in E2F targets, G2-M checkpoint, Myc targets, phagosome and ribosome-related processes. Collectively, these *in silico* validation approaches provide orthogonal evidence that distinct upstream molecular liabilities can trigger a shared downstream systems-level stress response.

RNA splicing abnormalities are increasingly recognized as a key regulatory mechanism in metabolic disease (Kaminska, 2025). Strikingly, beyond the shared upstream inflammatory signature, the most significant disease-specific pathway enrichment on the obesity side of our analysis was not further inflammation-related pathways, but RNA splicing and pre-mRNA processing, including the spliceosome and processing of capped intron-containing pre-mRNA. This finding suggests that widespread post-transcriptional dysregulation is a hallmark of the obese state, with pathological effects likely mediated through altered alternative splicing of key metabolic genes (Li et al., 2025; Fu et al., 2025). Notably, the inflammatory signature was not driven by shifts in immune cell proportions, as it remained evident after stratification by CD45 expression, underscoring its basis in cellular transcriptional reprogramming.

In stark contrast to the obesity-specific post-transcriptional splicing signature, and building on the shared upstream inflammatory background, the transcriptional signature on the depression side was overwhelmingly centred on the protein translation machinery. In Treg pseudo-bulk from MDD samples, the most significant pathway enrichments were for SRP-dependent co-translational protein targeting to membrane and the ribosome. This indicates a state of global translational dyshomeostasis in depression. Ribosomal dysfunction can directly impair the local synthesis of synaptic proteins, a process causally implicated in stress-induced depressive-like behaviours. Thus, depression may establish a central basis for metabolic dysregulation by reprogramming the translational machinery that underpins neuronal plasticity—a hypothesis that now warrants investigation in other relevant cell types and tissues.

Remarkably, our virtual cell knockout analyses revealed a unifying principle beneath these characteristic transcriptional divergences. Although the candidate gene sets on the depression side—including *ACAT1*, *CUL5*, *NPAT*, *FDFT1*, and *NEIL2*—and on the obesity side—*ACYP2*, *NT5C2*, *NT5DC2*, and *STK39*—showed no overlap, their knockout-induced perturbations repeatedly converged on a common set of downstream genes. These included regulators of cytoskeletal dynamics such as *STMN1*, *TUBA1B*, *TUBB*, and *CORO1A*; nuclear architecture and stress response genes such as *HMGN2*, *HMGB1*, *HMGB2*, *H2AFZ*, and *NEAT1*; and immune-related molecules such as *HLA-DRA*. Notably, perturbations of *NT5DC2* combined with either *ACAT1* or *FDFT1* exhibited the strongest cross-disease overlap, each sharing seven significantly perturbed genes. At the pathway level, these shared downstream genes were significantly enriched in phagosome and E2F targets, with additional convergence on tubulin folding, nuclear envelope dynamics, and apoptosis-related processes. The specificity analysis showed that the observed overlaps were 8.6- to 13.9-fold higher than random expectation (FDR <10^−5^), arguing against non-specific cellular stress as the sole explanation. This convergence suggests that distinct upstream molecular insults can trigger shared systems-level stress responses, manifesting in common pathological phenotypes at the interface of immune activation, cell cycle regulation, and cytoskeletal remodeling. Expanding on this cell-cycle and immune-metabolic interface, the specific perturbations of E2F targets, the G2-M checkpoint, and Myc targets are increasingly recognized to transcend the conventional paradigm of generic cellular stress, potentially operating as tailored molecular bridges within the metabolic-inflammatory-mood axis. Specifically, emerging evidence suggests that E2F targets may link clinical depression traits with hepatic lipid programming; previous studies indicate that blood E2F1 mRNA could serve as a distinct trait marker for depression and E2F2 inhibition has been shown to relieve depression-like behaviors, while hepatic E2F2 and E2F8 have been reported to modulate localized lipid storage and high-fat diet-induced lipogenesis (Takahashi et al., 2025; Xu et al., 2024; McGrory et al., 2019). Concurrently, accumulating data indicate that the G2-M checkpoint participates in regulating neural plasticity and metabolic inflammation; prior research has demonstrated that stress-induced glucocorticoids can arrest hippocampal neural precursors in the G1 phase to impair adult neurogenesis, while G2/M arrest is documented to cascade into downstream pro-inflammatory signaling interconnected with obesity complications (Peng et al., 2024; Patrício et al., 2021; Yang et al., 2010). Furthermore, Myc targets have been reported to exhibit a distinct dual identity, with literature suggesting they can serve as an intestinal metabolic disrupter that drives Cers4-mediated ceramide production and suppresses GLP-1 secretion in obesity, while simultaneously being implicated in fueling microglia activation and TNF-α/IL-6 release in depressive states (van Riggelen et al., 2010; Luo et al., 2021; Fu et al., 2022). Collectively, this functional cross-talk supports the hypothesis of an intrinsic, shared molecular web that coordinates cellular proliferation, tissue metabolism, and emotional control.

### Limitations

First, the bidirectional MR analysis suffered from asymmetric statistical power. The reverse direction (obesity to depression) had very low power (≤10%), so the null finding cannot be interpreted as evidence of absence of a causal effect. Therefore, our study does not establish a true biological asymmetry; rather, it highlights the need for larger, more powerful instruments for obesity in East Asian populations to more comprehensively clarify the potential reciprocal architecture of this comorbidity.

Second, a methodological asymmetry exists in our SMR analysis: depression used brain eQTL (PsychENCODE), while obesity used blood eQTL (CAGE consortium). This reflects the most biologically relevant tissue available for each trait, but it precludes direct quantitative comparison of eQTL effect sizes or gene-level significance between the two diseases. Our SMR analysis was therefore performed independently for each trait, and the resulting candidate genes should be interpreted as tissue-context-dependent hypotheses. The observed pattern of nominal genetic asymmetry is derived from bidirectional MR, which does not involve eQTL data. Furthermore, both eQTL panels are derived from European-ancestry individuals, whereas our GWAS summary statistics are from East Asian populations. This cross-population mismatch could introduce false positives due to differences in linkage disequilibrium structure. To evaluate this risk, we performed two sensitivity approaches: allele-frequency consistency was checked using a Japanese depression GWAS (Supplementary Table S3b), and quantitative LD was compared across populations for each SMR locus (Supplementary Table S3c). This LD analysis revealed locus-specific patterns: *NT5C2* showed highly similar LD between EUR and EAS panels (mean r^2^ = 1.000 vs. 0.977), whereas *ACYP2* and TMEM180 showed moderate-to-large differences (r^2^ difference = 0.166 and 0.269, respectively); *ACAT1* was not evaluable due to missing EAS LD data. Thus, while allele frequencies were broadly consistent, LD structure varied across loci—with *ACYP2*, TMEM180, and *ACAT1* requiring more cautious interpretation. Nonetheless, residual bias cannot be fully excluded, and the lack of direct ancestry-matched tissue eQTL data remains a limitation.

Third, the observed associations may be modulated by specific subtypes or life stages. For instance, fat mass, but not fat-free mass, is a causal risk factor for depression (Speed et al., 2019), and different adiposity axes confer opposing metabolic disease risks (Odoemelam et al., 2025). Obesity has been linked to the increased-appetite symptom of depression (Pistis et al., 2021), and *ADORA2A* polymorphisms are associated with sleep disturbances and attentional difficulties (Oliveira et al., 2019). Methodologically, the eQTL tissue sources differed between the depression and obesity sides, meaning the candidate gene strengths should not be compared directly. Additionally, the obesity single-cell dataset (GSE261178) exhibited tissue imbalance; therefore, results derived from this dataset—including the virtual knockout and shared pathway analyses—are exploratory in nature. The primary Mendelian randomization result for depression to obesity only reached nominal significance and should be viewed as hypothesis-generating. Furthermore, some studies in exclusively European cohorts still report bidirectional effects (Chen et al., 2023), suggesting potential ethnic heterogeneity or influences of phenotype definition that require validation in more diverse populations.

Fourth, as a purely computational study, our findings lack experimental validation. While our multi-omics integration provides a prioritized set of hypotheses and candidate targets, functional experiments—such as gene editing, pharmacological intervention, or animal model studies—are necessary to confirm causal relationships and biological mechanisms. Future work along these lines will be essential to translate our findings into mechanistic insights or therapeutic applications.

In conclusion, our findings refine our understanding of the traditional model of a fully symmetrical relationship between obesity and depression, by highlighting how statistical power asymmetries can shape causal inference, while providing hypothesis-generating evidence for a potential directional influence in which depression may contribute to metabolic susceptibility; expectedly, the reverse direction requires further investigation in better-powered studies. By identifying *ACYP2* as a prioritized obesity-side candidate for functional validation and ACAT1 as a hypothesis-generating anchor on the depression side—along with *ADORA2A*, *PDGFB*, and *FDFT1* as exploratory drug repurposing candidates—we offer a molecular framework that distinguishes genetically anchored causal candidates from translationally nominated nodes. The convergence of virtual knockout perturbations on shared downstream programs—including phagosome, E2F targets, and cytoskeletal dynamics—further suggests that distinct upstream insults may trigger common systems-level stress responses. This work highlights the importance of early metabolic risk monitoring in depression and lays a foundation for developing co-morbidity therapies targeting adenosine signaling, cholesterol synthesis, and immune-related pathways.

## Data Availability

The original contributions presented in the study are included in the article/Supplementary Material, further inquiries can be directed to the corresponding authors.
