# Supplementary material for: Genetic associations and candidate functional genes linking depression and obesity: a multi-omics integrative study
Source: Front Genet. 2026 Aug 7;17:1855173. doi: 10.3389/fgene.2026.1855173 (PMC13493084; doi:10.3389/fgene.2026.1855173)
Supplement: Supplementary file 1 [file DataSheet1.zip › Supplementary file/Figure Legend.docx]

### FigS1_MR_power

### Figure S1. Statistical power assessment for bidirectional Mendelian randomization (MR) analyses. The horizontal bar chart illustrates the approximate statistical power (%) to detect the observed Inverse Variance Weighted (IVW) effects for the bidirectional causal links between depression and obesity traits. The vertical dashed line marks the standard 80% statistical power threshold. Exact power estimates (63.7% for the forward direction and 6.9% for the reverse direction) are annotated adjacent to each bar.

FigS2_GWAS_meta_instrument_heterogeneity

Figure S2. Inter-study instrument heterogeneity in the GWAS meta-analyses.The bar plot displays the median I-squared (I^2^) statistics (%) evaluating the heterogeneity of genetic instruments extracted from the meta-analyzed summary statistics for depression and obesity with abdominal traits. Text annotations above each bar specify the total number of independent instrumental variables (n), the number of variants exhibiting moderate-to-high heterogeneity (I^2^ ≥50%), and the maximum recorded I^2^ value within each trait category.

FigS3_VKO_overlap_specificity

Figure S3. Downstream virtual knockout overlap (VKO) enrichment and specificity across phenotypes.

The horizontal bar plot depicts the downstream specificity analysis of identified priority risk genes. Bars represent the fold enrichment over random expectation for shared downstream targets between specific gene pairs (e.g., NT5DC2, STK39, ACYP2 interacting with metabolic/inflammatory downstream elements like ACAT1, FDFT1, CUL5). Text annotations adjacent to the bars indicate the absolute number of shared downstream molecules (shared), validating a robust and non-random cross-disease transcriptional intersection.

FigS4_NHANES_medication_sensitivity_Beta

Figure S4. NHANES medication sensitivity analysis for continuous adiposity traits.The forest plot presents the multivariable survey-weighted linear regression beta coefficients (with 95% confidence intervals) evaluating the cross-sectional association between depressive symptom burden (per 5-point increment in PHQ-9 score) and continuous adiposity phenotypes (BMI and waist circumference). For each trait, results are compared across three distinct analytical tiers: (1) the Base Covariates model, (2) the Medication-Adjusted model (further adjusting for antidepressants, glucose-lowering, lipid-lowering, and anti-obesity drugs), and (3) the Medication-User Excluded model (entirely excluding active prescription users). The vertical dashed line indicates the null effect (β = 0).

FigS5_NHANES_medication_sensitivity_OR

Figure S5. NHANES medication sensitivity analysis for categorical obesity outcomes.The forest plot displays the multivariable survey-weighted odds ratios (ORs with 95% confidence intervals) for the associations between clinically relevant depressive symptoms (PHQ-9 ≥10) and categorical disease states (general obesity and abdominal obesity). Association estimates are stratified across three stringent models: the Base Covariates model, the Medication-Adjusted model (incorporating four major drug classes as covariates), and the Medication-User Excluded model (restricting the sample to non-medication users). The vertical dashed line highlights the null risk threshold (OR = 1.0).

FigS6_CD45_key_pathway_heatmap

Figure S6. Heatmap of hallmark pathway perturbations across CD45-stratified and overall groups.

The heatmap illustrates the Normalized Enrichment Scores (NES) from gene set enrichment analysis (GSEA) for core inflammatory, metabolic, and cell-cycle pathways across four designated cell-type or phenotypic clusters: Obesity overall, depression-associated regulatory T cells (depression_Treg_overall), obesity-derived CD45-negative cells, and obesity-derived CD45-positive immune populations. The numeric labels within each tile indicate the exact NES value, with the color gradient ranging from blue (downregulation) to red (upregulation).
