# Supplementary material for: Genetic associations and candidate functional genes linking depression and obesity: a multi-omics integrative study
Source: Front Genet. 2026 Aug 7;17:1855173. doi: 10.3389/fgene.2026.1855173 (PMC13493084; doi:10.3389/fgene.2026.1855173)
Supplement: Supplementary file 1 [file DataSheet1.zip › Supplementary file/Supplementary Table.docx]

**Supplementary Table**

**Supplementary Table S1. Power calculations for bidirectional Mendelian randomization between depression and obesity**

| **Direction** | **MR method** | **Power to detect observed effect (own β)** | **Power to detect effect size observed in the opposite direction** | **Minimum detectable effect size (β) at 80% power** | **Mean F-statistic** | **Variance explained (R²)** |
| --- | --- | --- | --- | --- | --- | --- |
| Depression to obesity | IVW | 63.7% | 40.70% | 0.0555 | 32.25 | 0.115 |
| Depression to obesity | Weighted median | 55.1% | 28.80% | 0.0684 | 32.25 | 0.115 |
| Depression to obesity | MR Egger | 14.2% | 15.00% | 0.1045 | 32.25 | 0.115 |
| Depression to obesity | Simple mode | 28.2% | 16.00% | 0.1 | 32.25 | 0.115 |
| Depression to obesity | Weighted mode | 20.6% | 12.70% | 0.1189 | 32.25 | 0.115 |
| Obesity to depression | IVW | 6.9% | 8.40% | 0.2382 | 41.69 | 0.074 |
| Obesity to depression | Weighted median | 16.4% | 6.90% | 0.3153 | 41.69 | 0.074 |
| Obesity to depression | MR Egger | 18.5% | 5.50% | 0.6451 | 41.69 | 0.074 |
| Obesity to depression | Simple mode | 8.2% | 5.30% | 0.7523 | 41.69 | 0.074 |
| Obesity to depression | Weighted mode | 9.7% | 5.30% | 0.8159 | 41.69 | 0.074 |

*Power calculations were performed using a normal approximation based on the observed IVW β and its standard error, assuming α = 0.05. For each direction, we calculated (i) the power to detect the observed effect size (using its own β and SE), (ii) the power to detect the effect size observed in the opposite direction, and (iii) the minimum detectable absolute effect size (β) at 80% power. The approximate variance explained by the instruments (R²) was derived from allele frequencies and effect sizes.

**Supplementary Table S2a. Contribution of individual instrumental variants to heterogeneity in forward MR (depression to obesity)**

| **SNP** | **Wald ratio (β)** | **Q contribution (%)** | **Cumulative Q contribution (%)** |
| --- | --- | --- | --- |
| 18:68034196:c:t | -0.04114 | 47.03 | 47.03 |
| 2:180935661:c:t | 0.12968 | 46.9 | 93.93 |
| 22:39372088:a:g | 0.02622 | 3.43 | 97.36 |
| 8:41890793:c:t | 0.06391 | 1.45 | 98.81 |
| 22:21957989:a:g | 0.05417 | 0.53 | 99.34 |
| 11:108341864:c:t | 0.0533 | 0.34 | 99.68 |
| 5:151925726:c:t | 0.03483 | 0.33 | 100.01 |

*Shown are all seven instrumental variables (SNPs) used in the forward MR analysis. Wald ratio represents the causal effect estimate for each SNP (β). Q contribution (%) indicates the percentage each SNP contributes to the total Cochran’s Q statistic. Cumulative Q contribution (%) is the running sum. The overall Cochran’s Q test for heterogeneity was non‑significant (P = 0.278), indicating no evidence of heterogeneity. Percentages may not sum exactly to 100% due to rounding.

**Supplementary Table S2b. Contribution of individual instrumental variants to heterogeneity in forward MR (depression to obesity)**

| **SNP** | **Wald ratio (β)** | **Q contribution (%)** | **Cumulative Q contribution (%)** |
| --- | --- | --- | --- |
| 6:138020871:c:t | -1.46425 | 7.59 | 7.59 |
| 6:20686878:c:t | 1.09521 | 6.36 | 13.95 |
| 12:130646060:a:c | -1.47343 | 6.23 | 20.18 |
| 4:45182527:a:g | -1.1489 | 5.44 | 25.62 |
| 18:19888005:c:t | 1.51783 | 5.33 | 30.95 |
| 2:181517642:c:t | 1.29096 | 4.97 | 35.92 |
| 18:57852587:c:t | 0.66692 | 4.76 | 40.68 |
| 1:117876437:g:t | -1.17267 | 4.61 | 45.29 |
| 9:97094721:c:t | 1.30546 | 4.45 | 49.74 |
| 5:122750847:c:t | -1.10095 | 4.09 | 53.83 |
| 17:55331120:a:g | 1.29761 | 3.91 | 57.74 |
| 4:5743053:a:g | 1.18999 | 3.79 | 61.53 |
| 10:104958900:a:g | -0.97883 | 3.76 | 65.29 |
| 20:34634914:g:t | -1.06559 | 3.07 | 68.36 |
| 1:19954816:a:g | -0.96716 | 3.07 | 71.43 |
| 2:54433327:a:g | -0.94236 | 2.95 | 74.38 |
| 12:109925484:a:g | 0.64279 | 2.9 | 77.28 |
| 8:34472690:c:t | 0.92694 | 2.5 | 79.78 |
| 2:632904:a:g | -0.44659 | 2.1 | 81.88 |
| 7:26100558:a:g | -0.74426 | 1.99 | 83.87 |
| 2:25109302:a:g | -0.64741 | 1.62 | 85.49 |
| 15:99443161:a:g | 0.75278 | 1.38 | 86.87 |
| 1:54798752:c:t | -0.64975 | 1.19 | 88.06 |
| 3:173045367:c:t | -0.49229 | 0.81 | 88.87 |
| 2:169129145:g:t | -0.36367 | 0.8 | 89.67 |
| 9:138380863:c:t | 0.5522 | 0.75 | 90.42 |
| 4:20321977:c:g | -0.41022 | 0.75 | 91.17 |
| 16:53845487:a:c | 0.28925 | 0.71 | 91.88 |
| 10:77437044:c:t | 0.53407 | 0.7 | 92.58 |
| 17:43589440:c:t | 0.42953 | 0.69 | 93.27 |
| 11:117958179:a:t | -0.42811 | 0.65 | 93.92 |
| 14:63149261:g:t | 0.48897 | 0.57 | 94.49 |
| 18:29959863:c:g | 0.40591 | 0.54 | 95.03 |
| 1:177873210:g:t | 0.27231 | 0.52 | 95.55 |
| 19:46221726:a:g | 0.30539 | 0.5 | 96.05 |
| 4:94487317:a:g | -0.38555 | 0.5 | 96.55 |
| 6:50849459:c:t | -0.29096 | 0.47 | 97.02 |
| 20:60649412:c:t | -0.51022 | 0.46 | 97.48 |
| 7:138817193:c:t | 0.3938 | 0.44 | 97.92 |
| 16:20248599:a:g | -0.29126 | 0.39 | 98.31 |
| 6:156705461:a:g | -0.27034 | 0.27 | 98.58 |
| 6:73851837:a:c | -0.20535 | 0.17 | 98.75 |
| 1:32233572:g:t | 0.30504 | 0.17 | 98.92 |
| 3:52893465:c:t | 0.204 | 0.16 | 99.08 |
| 6:34225269:c:t | -0.21838 | 0.16 | 99.24 |
| 3:84636825:a:g | 0.25607 | 0.15 | 99.39 |
| 5:133861663:c:t | 0.22512 | 0.15 | 99.54 |
| 11:27670108:c:t | -0.10763 | 0.13 | 99.67 |
| 11:8605775:a:g | 0.22552 | 0.11 | 99.78 |
| 13:54084032:a:g | 0.16856 | 0.08 | 99.86 |
| 3:185454027:a:g | -0.10625 | 0.06 | 99.92 |
| 8:89758027:g:t | -0.0393 | 0.05 | 99.97 |
| 2:242510982:a:g | 0.15389 | 0.03 | 100 |
| 6:110219629:a:c | 0.13055 | 0.03 | 100.03 |
| 11:65689304:a:g | 0.09055 | 0.01 | 100.04 |
| 5:95856459:g:t | 0.00675 | 0 | 100.04 |
| 4:183524210:c:t | 0.00853 | 0 | 100.04 |
| 15:51162261:c:t | 0.01549 | 0 | 100.04 |
| 2:81933734:c:t | 0.03255 | 0 | 100.04 |

*All 59 instrumental variables (SNPs) used in the reverse MR analysis are listed in descending order of their contribution to the total Cochran’s Q statistic. Wald ratio is the SNP‑specific causal estimate (β). Q contribution (%) is the percentage of the total Q statistic attributable to each SNP. Cumulative Q contribution (%) is the running sum. The overall Cochran’s Q test showed significant heterogeneity (P = 0.024). The first two SNPs (6:138020871:c:t and 6:20686878:c:t) together contributed 13.95% of the total Q. Due to rounding of individual percentages to two decimal places, the cumulative sum may slightly deviate from 100% (final sum 100.04%).

**Supplementary Table S3a. Exploratory SMR results using a single Japanese depression GWAS (GCST006644) with PsychENCODE brain eQTL.**

| **Gene** | **Chr** | **Lead SNP** | **β (SMR)** | **Se (SMR)** | **P (SMR)** | **P (GWAS)** |
| --- | --- | --- | --- | --- | --- | --- |
| KDELC2 | 11 | rs10890858 | -0.152764 | 0.104947 | 0.1455 | 0.1425 |
| CUL5 | 11 | rs10890805 | 0.914399 | 1.06287 | 0.3896 | 0.3857 |
| AF131215.9 | 8 | rs4841495 | 0.259902 | 0.343275 | 0.449 | 0.4478 |
| NEIL2 | 8 | rs9650662 | 0.128075 | 0.240524 | 0.5944 | 0.5927 |
| FAM167A | 8 | rs1347410 | -0.069271 | 0.276694 | 0.8023 | 0.8022 |

*This analysis was performed as a sensitivity QC rerun using a single Japanese depression GWAS (GCST006644) with PsychENCODE brain eQTL. HEIDI was disabled. P (SMR) values are nominal (not adjusted for multiple comparisons); no gene reached nominal significance (all P (SMR) > 0.14). P (GWAS) is the GWAS association P value for the lead SNP. This table does not replace the primary SMR/HEIDI findings from the five‑GWAS meta‑analysis.

**Supplementary Table S3b. Comparison of allele‑frequency QC between PsychENCODE brain eQTL and CAGE blood eQTL using a Japanese depression GWAS (GCST006644).**

| **Analysis** | **GWAS source** | **eQTL source** | **Tissue** | **Genome build** | **Allele-matched SNPs** | **SNPs with frequency difference >0.20** | **Percentage failed** | **Default SMR frequency QC** |
| --- | --- | --- | --- | --- | --- | --- | --- | --- |
| Primary depression-side SMR QC | GCST006644 Japanese depression GWAS | PsychENCODE PEER50 brain eQTL | Brain | hg19/GRCh37 | 4155 | 0 | 0.00% | Passed |
| Blood eQTL sensitivity QC | GCST006644 Japanese depression GWAS | CAGE blood eQTL | Blood | hg19/GRCh37 | 20249 | 5492 | 27.12% | Failed |

*The default SMR allele‑frequency stop threshold is a difference >0.20. “Allele‑matched SNPs” refers to the number of variants successfully aligned between the eQTL and GWAS after harmonization. “Percentage failed” indicates the proportion of matched SNPs with frequency difference >0.20. QC outcome is determined by whether any SNP exceeds the threshold (fail) or not (pass).

**Supplementary Table S3c. Cross-population linkage disequilibrium comparison for SMR-prioritized gene loci between European and East Asian reference panels.**

| **Trait** | **Gene** | **SMR top eQTL SNP** | **Regional GWAS lead SNP** | **EUR subpopulations (n)** | **EAS subpopulations (n)** | **EUR mean r2** | **EAS mean r2** | **Absolute mean r2 difference** | **Interpretation** |
| --- | --- | --- | --- | --- | --- | --- | --- | --- | --- |
| Depression | ACAT1 | rs3741054 | rs7934719 | 5 | 0 | 0.151 | NA | NA | Not evaluable |
| Depression | CUL5 | rs7102368 | rs7934719 | 1 | 0 | 0.078 | NA | NA | Not evaluable |
| Depression | KDELC2 | rs74822968 | rs7934719 | 5 | 4 | 0.107 | 0.125 | 0.018 | Similar |
| Depression | NEIL2 | rs113948051 | rs12545407 | 0 | 0 | NA | NA | NA | Not evaluable |
| Depression | UBE2L3 | rs4821104 | rs138822582 | 0 | 4 | NA | 0.085 | NA | Not evaluable |
| Depression | YDJC | rs4821104 | rs138822582 | 0 | 4 | NA | 0.085 | NA | Not evaluable |
| Depression | FAM167A | rs1347410 | rs12545407 | 0 | 0 | NA | NA | NA | Not evaluable |
| Depression | AF131215.2 | rs2572408 | rs12545407 | 0 | 0 | NA | NA | NA | Not evaluable |
| Depression | AF131215.9 | rs2099456 | rs12545407 | 0 | 0 | NA | NA | NA | Not evaluable |
| Obesity | NT5C2 | rs11191607 | rs112913898 | 5 | 5 | 1 | 0.977 | 0.023 | Similar |
| Obesity | ACYP2 | rs7592069 | rs1215146 | 5 | 5 | 0.153 | 0.318 | 0.166 | Moderate |
| Obesity | NT5DC2 | rs13621 | rs6798941 | 5 | 5 | 0.287 | 0.426 | 0.139 | Moderate |
| Obesity | TMEM180 | rs77335224 | rs112913898 | 5 | 5 | 0.862 | 0.593 | 0.269 | Large |
| Obesity | STK39 | rs4668044 | rs1955337 | 5 | 5 | 0.199 | 0.186 | 0.013 | Similar |
| Obesity | MAPK3 | rs7201780 | rs9939774 | 5 | 5 | 0.472 | 0.629 | 0.157 | Moderate |
| Obesity | DMPK | rs10404730 | rs11881883 | 2 | 5 | 0.072 | 0.26 | 0.188 | Moderate |
| Obesity | ITIH4 | rs11717749 | rs6798941 | 1 | 0 | 0.145 | NA | NA | Not evaluable |
| Obesity | TMEM110 | rs11708390 | rs6798941 | 0 | 0 | NA | NA | NA | Not evaluable |
| Obesity | SAE1 | rs309190 | rs2287842 | 1 | 0 | 0.055 | NA | NA | Not evaluable |

*LD was queried using the Ensembl REST API with the 1000 Genomes Phase 3 reference panel. Coordinates were based on GRCh37/hg19. EUR subpopulations: CEU (Utah Residents with Northern and Western European Ancestry), FIN (Finnish in Finland), GBR (British in England and Scotland), IBS (Iberian Populations in Spain), TSI (Toscani in Italia). EAS subpopulations: CDX (Chinese Dai in Xishuangbanna), CHB (Han Chinese in Bejing), CHS (Han Chinese South), JPT (Japanese in Tokyo), KHV (Kinh in Ho Chi Minh City). GWAS variants were mapped to rsIDs using the Ensembl GRCh37 overlap endpoint with allele matching when available. Among 19 unique SMR gene loci, EUR-EAS mean r² comparison was available for 8 loci (KDELC2, NT5C2, ACYP2, NT5DC2, TMEM180, STK39, MAPK3, DMPK) and unavailable for 11 loci because the Ensembl/1000G LD endpoint did not return pairwise LD data for one or both ancestry groups. Of the evaluable loci, 3 showed similar LD (difference <0.05), 4 showed moderate differences (0.05 to <0.20), and 1 showed a large difference (≥0.20). These results support an ancestry-sensitive interpretation of SMR/HEIDI prioritized loci rather than a blanket assumption of identical LD structure across populations.

**Supplementary Table S4. Specificity analysis of downstream gene overlaps in cross‑disease virtual knockout pairs.**

| **obesity ko gene** | **Depression ko gene** | **Shared (n)** | **Expecte (random)** | **Observed/expected ratio** | **FDR** | **Shared genes** |
| --- | --- | --- | --- | --- | --- | --- |
| NT5DC2 | ACAT1 | 7 | 0.53 | 13.14 | 5.84E-06 | H2AFZ;HMGB1;HMGB2;HMGN2;STMN1;TUBA1B;TUBB |
| NT5DC2 | FDFT1 | 7 | 0.75 | 9.38 | 3.84E-05 | CORO1A;H2AFZ;HMGB2;HMGN2;STMN1;TUBA1B;TUBB |
| NT5C2 | FDFT1 | 4 | 0.3 | 13.2 | 9.78E-04 | CORO1A;STMN1;TUBA1B;TUBB |
| NT5C2 | ACAT1 | 3 | 0.22 | 13.86 | 5.09E-03 | STMN1;TUBA1B;TUBB |
| STK39 | ACAT1 | 2 | 0.23 | 8.58 | 8.51E-02 | HMGN2;STMN1 |
| STK39 | FDFT1 | 2 | 0.33 | 6.13 | 1.34E-01 | HMGN2;STMN1 |
| NT5C2 | CUL5 | 1 | 0.24 | 4.2 | 4.87E-01 | NEAT1 |
| ACYP2 | ACAT1 | 1 | 0.25 | 4 | 4.87E-01 | HLA-DRA |
| STK39 | CUL5 | 1 | 0.26 | 3.9 | 4.87E-01 | NEAT1 |
| ACYP2 | CUL5 | 1 | 0.27 | 3.64 | 4.87E-01 | NEAT1 |
| ACYP2 | FDFT1 | 1 | 0.35 | 2.86 | 5.45E-01 | CORO1A |

*Hypergeometric test was used to assess whether observed overlaps are greater than expected by random set‑size. Expected (random) is the expected number of shared genes under the null hypothesis of independence, calculated from the hypergeometric distribution. Observed/expected ratio = shared_n / expected_random. FDR was controlled using the Benjamini‑Hochberg method. Only pairs with at least one shared gene are shown; pairs with zero shared genes are omitted.

**Supplementary Table S5. Hallmark pathway enrichment in overall, CD45+ and CD45‑ cells of the obesity dataset, and in depression Tregs.**

| **Group** | **Pathway** | **NES** | **FDR** |
| --- | --- | --- | --- |
| Obesity (overall) | TNF-alpha Signaling via NF-kB | 2.66 | 6.55E-17 |
| Obesity (overall) | Inflammatory Response | 2.294 | 1.79E-10 |
| Obesity (overall) | Interferon Alpha Response | 1.919 | 9.22E-05 |
| Obesity (overall) | Interferon Gamma Response | 2.018 | 8.30E-07 |
| Obesity (overall) | E2F Targets | -1.098 | 0.257 |
| Obesity (overall) | G2-M Checkpoint | -1.432 | 0.015 |
| Obesity (overall) | Myc Targets V1 | 0.763 | 0.974 |
| Obesity (overall) | Oxidative Phosphorylation | 1.731 | 0.0003 |
| Obesity (CD45+) | TNF-alpha Signaling via NF-kB | 3.128 | 1.43E-30 |
| Obesity (CD45+) | Inflammatory Response | 2.818 | 4.36E-20 |
| Obesity (CD45+) | Interferon Alpha Response | 0.956 | 0.658 |
| Obesity (CD45+) | Interferon Gamma Response | 1.681 | 0.00037 |
| Obesity (CD45+) | E2F Targets | -1.003 | 0.578 |
| Obesity (CD45+) | G2-M Checkpoint | -1.106 | 0.276 |
| Obesity (CD45+) | Myc Targets V1 | 0.832 | 0.874 |
| Obesity (CD45+) | Oxidative Phosphorylation | 1.878 | 3.04E-06 |
| Obesity (CD45-) | TNF-alpha Signaling via NF-kB | -1.155 | 0.32 |
| Obesity (CD45-) | Inflammatory Response | -1.522 | 0.0088 |
| Obesity (CD45-) | Interferon Alpha Response | 2.444 | 2.36E-09 |
| Obesity (CD45-) | Interferon Gamma Response | 1.83 | 4.89E-05 |
| Obesity (CD45-) | E2F Targets | -0.925 | 0.795 |
| Obesity (CD45-) | G2-M Checkpoint | -1.438 | 0.024 |
| Obesity (CD45-) | Myc Targets V1 | -0.71 | 0.997 |
| Obesity (CD45-) | Oxidative Phosphorylation | -0.828 | 0.997 |
| Depression (Tregs) | TNF-alpha Signaling via NF-kB | 2.49 | 5.17E-13 |
| Depression (Tregs) | Inflammatory Response | 1.294 | 0.153 |
| Depression (Tregs) | Interferon Alpha Response | 1.626 | 0.0146 |
| Depression (Tregs) | Interferon Gamma Response | 1.094 | 0.472 |
| Depression (Tregs) | E2F Targets | -0.823 | 0.966 |
| Depression (Tregs) | G2-M Checkpoint | 1.071 | 0.534 |
| Depression (Tregs) | Myc Targets V1 | 1.834 | 0.000136 |
| Depression (Tregs) | Oxidative Phosphorylation | 1.597 | 0.0047 |

*NES: normalized enrichment score from fgsea. FDR: Benjamini‑Hochberg adjusted P value. Positive NES indicates upregulation in disease vs. control (obesity: obese vs. lean; depression: MDD vs. control); negative NES indicates downregulation. Groups: Obesity (overall) – all cells; Obesity (CD45+) – immune cells; Obesity (CD45-) – non‑immune cells; Depression (Tregs) – Tregs from MDD vs. control.
